# Extraction, structural characterization, chemical modification and anti-inflammatory activity of polysaccharides from *Ceratocarpus arenarius* L.

**DOI:** 10.3389/fnut.2026.1785022

**Published:** 2026-02-26

**Authors:** Junlong Wang, Yuhao Cui, Jie Li, Yonggang Lin, Wei Feng

**Affiliations:** Key Laboratory of Yili Kazakh Medicinal Resources Excavation and Modernization Research, School of Chemistry and Chemical Engineering, Yili Normal University, Yining, Xinjiang, China

**Keywords:** *Ceratocarpus arenarius* L., polysaccharide, structural characterization, chemical modification, anti-inflammatory activity

## Abstract

The extraction process for crude polysaccharides from *Ceratocarpus arenarius* L. was optimized using response surface methodology (RSM). A major polysaccharide fraction with high purity, designated CAP, was isolated from the crude polysaccharides using DEAE-650 M and Sephadex G-75 column chromatography. Its structure was comprehensively characterized using FT-IR, partial acid hydrolysis, peroxide oxidation, Smith degradation, methylation analysis, and NMR analysis. Subsequently, carboxymethylated (CAP-C), acetylated (CAP-A), sulfated (CAP-S), and phosphorylated (CAP-P) derivatives of CAP were prepared. Their structures were characterized using techniques, including HPSEC, GC, UV, FT-IR, Congo red assay, SEM, XRD, and thermal stability analysis. The results of the RSM optimization indicated that the optimal extraction conditions were as follows: time 43 min, ultrasonic power 310 W, liquid–solid ratio 21:1 mL/g, temperature 62 °C. The crude polysaccharide yield obtained under these conditions was 19.09 ± 0.12%. CAP is primarily composed of pyranose rings linked by α-glycosidic bonds. Its backbone primarily consists of →3)-Xyl*p*-(1→, →3)-Gal*p*-(1→, Gal*p*-(1→, and →2,4)-Gal*p*-(1→ glycosidic bonds, while the terminal residues of the side chains are mainly T-Xyl*p* and T-Rha*p*. Structural modification significantly altered the monosaccharide molar ratio, molecular weight, viscosity, solubility, surface morphology, crystalline characteristics, and thermodynamic properties of CAP and its derivatives. Anti-inflammatory activity studies revealed that different modification methods differentially enhanced the polysaccharide bioactivity. However, all derivatives could effectively suppress LPS-induced inflammatory responses by regulating the production of nitric oxide (NO), prostaglandin E2 (PGE_2_), interleukin-6 (IL-6), interleukin−1β (IL-1β), and tumor necrosis factor-α (TNF-α). These findings suggest that CAP and its derivatives have potential applications in the functional food and pharmaceutical industries.

## Graphical abstract

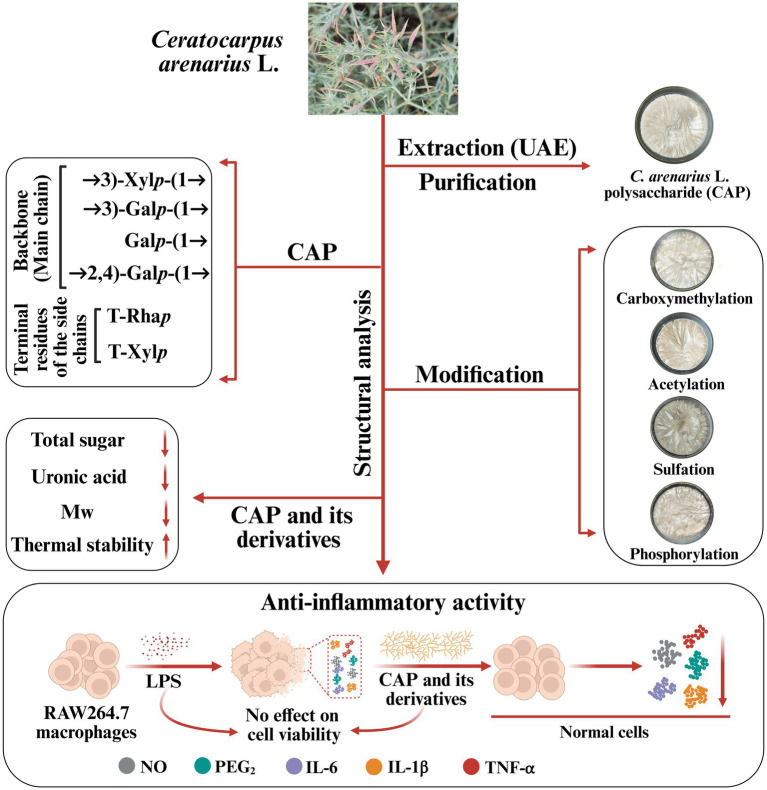

## 1 Introduction

Inflammation is a complex pathological process and the body’s defensive response to harmful stimuli, such as pathogens, parasites, or physical and chemical factors (1). However, excessive or persistent inflammatory responses promote the development of chronic diseases (2). World Health Organization (WHO) survey data show that approximately 60% of global mortality is related to diseases associated with chronic inflammation, making inflammation research a key area in medicine and public health worldwide (3). As key pro-inflammatory cells, macrophages constitute the first line of immune defense through the release of mediators such as NO, PGE_2_, and TNF-α (4). However, their over-activation can lead to the accumulation of pro-inflammatory cytokines, which in turn can trigger diseases such as asthma, diabetes, and rheumatoid arthritis (5). Although conventional anti-inflammatory drugs such as NSAIDs are effective in relieving symptoms, long-term use may trigger side effects such as gastrointestinal damage and immunosuppression (6). Consequently, the development of non-toxic, low side effect, natural active ingredients capable of modulating macrophage activation has become an important strategy for the treatment of inflammation-related diseases.

In recent years, natural active ingredients derived from medicinal plants have received widespread attention in the field of disease treatment and health maintenance due to their unique pharmacological effects, low toxicity, and low side effects (7). *Ceratocarpus arenarius* L. (*C. arenarius* L.) is a representative of the quinoa family of medicinal plants, which is mainly distributed in the arid regions of Central Asia, West Asia, Eastern Europe and Northwest China, and its abundant polysaccharides, flavonoids and alkaloids constitute the material basis of the pharmacological effects of *C. arenarius* L. (8). The Chinese Pharmacopeia records that *C. arenarius* L. has pharmacological effects such as anti-inflammatory, analgesic, hemostatic and regulating the digestive system (9). Numerous studies have shown that natural compounds such as polysaccharides, polyphenols and flavonoids have unique biological activities in anti-inflammation, among which polysaccharides are particularly prominent in protecting cells from inflammatory damage, and at the same time, due to their high efficiency and non-toxicity, they are considered the most valuable anti-inflammatory candidates for research (10–12).

Polysaccharides are a class of natural macromolecular compounds formed by the polymerization of monosaccharides, which are widely found in plants, animals, and microorganisms (13). Modern pharmacological studies have shown that polysaccharides have a variety of biological activities such as antioxidant (14), anti-tumor (15), anti-inflammatory (16), anti-aging (17), and hypoglycemic (18). However, the problems of high viscosity, low solubility and weak bioactivity of natural polysaccharides have limited their practical applications (19). Structural modification of polysaccharides is to improve the physicochemical properties and biological activities of polysaccharides through the introduction of new groups, and even make them endowed with new activities (20). However, the structural modification of polysaccharides is not as flexible as the structural modification of small molecules, and at present, there are mainly carboxymethylation (21), acetylation (22), sulfation (23), phosphorylation (24) modification, and other methods. Li et al. (25) showed that the antitumor activity of carboxymethylation modified *Corn bran* polysaccharides was significantly enhanced, and the inhibition of A549 and HepG-2 tumor cells by the modified polysaccharides was increased by 11.18 and 16.24%, respectively, as compared with the natural polysaccharides. In addition, Wu et al. (26) reported that *Sargassum cristaefolium* polysaccharide significantly inhibited LPS-induced NO secretion (74.1% inhibition rate) in RAW264.7 macrophages by introducing sulfate groups, demonstrating a superior anti-inflammatory effect than the natural polysaccharide. Despite the long history of medicinal use of *C. arenarius* L., studies on the extraction and structural characterization of its polysaccharide constituents, and the effect of their different chemical modifications on bioactivity, have not been reported.

In the present study, *C. arenarius* L. crude polysaccharides were extracted by an ultrasound-assisted method, and after isolation and purification, a high-purity polysaccharide fraction (CAP) was obtained. The structure of CAP was resolved by FT-IR, partial acid hydrolysis, peroxide oxidation, Smith degradation, methylation analysis, and NMR analysis. Subsequently, carboxymethylated, acetylated, sulfated, and phosphorylated derivatives were prepared by structural modification, and the structural features and physicochemical properties of the four derivatives were compared by using UV, HPSEC, GC, Congo red assay, SEM, XRD, and thermal stability analysis. In addition, the effects of different chemically modified polysaccharides on anti-inflammatory activities were evaluated. This study not only fills the research gap of CAP and its derivatives, but also provides a scientific basis for screening the modified modifications of highly active polysaccharides.

## 2 Materials and methods

### 2.1 Materials and reagents

The *C. arenarius* L. was collected from the Xinjiang Yili region of China and identified by Prof. Zhang Wei from the Yili Normal University. Currently deposited in the Key Laboratory of Natural Products (No. WJL-13).

DEAE Cellulose-650 M, Sephadex G-75, and dextrans (T-10, T-40, T-70, T-200, and T-500 kDa) were purchased from Shanghai Yuanye Biological Technology Co., LTD. Standard monosaccharides (fucose, rhamnose, arabinose, xylose, mannose, glucose, and galactose) were purchased from Resource Leaf Biotechnology Co., LTD. Deuterium oxide (99.9% D, catalog number, D807644) was purchased from Macklin Biochemical Technology Co., LTD. Macrophage cell line RAW 264.7 was purchased from Cell Resource Center, Institute of Basic Medical Sciences, Chinese Academy of Medical Sciences. Lipopolysaccharide (LPS) was purchased from Beijing Solarbio Biotechnology Co., LTD. All other chemicals used in the work were of analytical grade and purchased from local suppliers.

### 2.2 Extraction of crude polysaccharides

#### 2.2.1 Pretreatment

The dried leaves of *C. arenarius* L. were crushed and passed through a 40-mesh sieve, then degreased with petroleum ether (1:20, w/v) under reflux at 70 °C for 24 h. Subsequently, after natural air-drying, the material was depigmented with anhydrous ethanol (1:20, w/v) under reflux at 78 °C for 24 h, and air-dried to obtain the degreased and depigmented *C. arenarius* L. powder.

#### 2.2.2 Single-factor design

The sample powder was extracted using an ultrasonic cell disruptor (JY92-IIDN, Scientz, China). The effects of extraction time (10 ~ 50 min), ultrasonic power (100 W ~ 500 W), liquid–solid ratio (10:1 mL/g ~ 50:1 mL/g) and extraction temperature (30 °C ~ 70 °C) on the yield were investigated. In this experiment, each trial only varied one of the aforementioned factors until all necessary single-factor experiments were completed. After extraction, the solution was treated with the Sevage method to remove proteins (27). Subsequently, anhydrous ethanol was slowly added to adjust the final concentration to 80%. The mixture was then placed at 5 °C for 24 h and allowed to settle. After centrifugation (5,000 rpm, 10 min), the precipitate was collected. The crude polysaccharides were obtained after freeze-drying (−70 °C, 48 h). The yield was calculated according to Equation 1:


$$Y(\%)=\frac{m}{M}\times 100\%$$ (1)

Where *Y*-the yield of crude polysaccharides (%), *m*-the weight of CA (mg), and *M*-the weight of material for extraction (mg).

#### 2.2.3 Box–Behnken design

Based on the results of single-factor experiments, Box–Behnken design (BBD) and response surface methodology (RSM) were further employed to optimize four factors: extraction time (A), ultrasound power (B), liquid–solid ratio (C), and temperature (D) (28). The aim was to maximize the yield of crude polysaccharides. Four factors with three levels were established, where each factor was coded as −1 (low value), 0 (middle value), and +1 (high value); total crude polysaccharide yield (%) was considered the response variable. A total of 29 experiments were conducted, including 24 analysis points and 5 replication points. See Table 1 for details.

**Table 1:** Variables and their levels used in BBD and RSM.

Run	A: Time (min)	B: Power (W)	C: Liquid–solid ratio (mL/g)	D: Temperature (°C)	Y: Yield (%)
1	−1	0	−1	0	14.03
2	−1	1	0	0	14.69
3	0	0	−1	−1	15.53
4	−1	−1	0	0	14.88
5	0	0	0	0	18.83
6	0	−1	0	1	16.31
7	1	1	0	0	14.49
8	−1	0	0	1	15.95
9	0	−1	1	0	16.23
10	0	1	0	1	15.29
11	0	0	0	0	18.79
12	1	−1	0	0	16.02
13	0	1	−1	0	14.58
14	−1	0	1	0	17.05
15	0	0	0	0	18.85
16	0	0	−1	1	15.98
17	0	0	1	1	16.7
18	0	−1	0	−1	16.19
19	0	0	0	0	18.81
20	0	−1	−1	0	17.83
21	0	1	0	−1	14.88
22	1	0	1	0	15.78
23	1	0	−1	0	13.78
24	−1	0	0	−1	15.89
25	0	0	0	0	18.84
26	0	0	1	−1	16.51
27	1	0	0	−1	15.82
28	0	1	1	0	15.05
29	1	0	0	1	15.59

### 2.3 Isolation and purification of crude polysaccharides

The crude polysaccharide (1 g) was dissolved in a small volume of distilled water. The supernatant was then loaded onto a DEAE-650 M cellulose column (52 × 360 mm) and eluted sequentially with three column volumes of distilled water, 0.2 M, 0.4 M, and 0.6 M NaCl solutions at a flow rate of 1.0 mL/min (4.0 mL per tube). The elution process was monitored by the anthrone-sulfuric acid method (29). Fractions corresponding to the same peak in the elution curve were combined, then dialyzed (MWCO: 3500 Da), and freeze-dried (−70 °C, 48 h) to yield a preliminarily purified polysaccharide fraction. Subsequently, the sample was loaded onto a Sephadex G-75 column (16 × 800 mm), and further purified with distilled water (three column volumes) at a flow rate of 0.4 mL/min (2.0 mL per tube). The same polysaccharide fractions were collected and combined, followed by dialysis and freeze-drying to obtain a high-purity polysaccharide fraction, designated CAP.

### 2.4 Structural characterization of CAP

#### 2.4.1 Monosaccharide composition determination

The monosaccharide composition of polysaccharide samples (CAP and its derivatives) was determined by pyridine-acetic anhydride method and GC (GC-8890, Agilent, United States) (HP-5 column: 30 m × 0.32 mm × 0.25 μm, FID detector) (30). In this experiment, a 5 mg sample of each polysaccharide was weighed, mixed separately with 0.5 mL of pyridine and 2 mL of 2 M trifluoroacetic acid (TFA), and hydrolyzed at 100 °C for 2 h (12). After cooling to room temperature, the solution was washed repeatedly (5 times) with methanol (2 mL) and dried to remove residual TFA. The hydrolysis products were then reduced with sodium borohydride (NaBH₄) solution (10 mg/mL) at room temperature for 90 min, followed by the addition of 0.5 mL acetic anhydride for acetylation reaction at 100 °C for 20 min. After the reaction, the supernatant was blown dry with nitrogen (N_2_), and 1 mL of dichloromethane was added. Monosaccharide standards, including fucose (Fuc), rhamnose (Rha), arabinose (Ara), xylose (Xyl), mannose (Man), glucose (Glc) and galactose (Gal), do not need to be hydrolyzed, and the remaining steps of derivatization are performed as described above. Monosaccharides were identified by comparing their chromatograms with reference monosaccharides. Relative molar ratios were calculated using area normalization (31). The final sample was analyzed using the following procedure: detector temperature and injector temperature at 260 °C, initial temperature held at 160 °C for 5 min, followed by a temperature increase to 190 °C at a rate of 3 °C/min over 5 min.

#### 2.4.2 Partial acid hydrolysis

Based on the characteristic that side-chain glycosidic bonds are more susceptible to acidic influences than main-chain glycosidic bonds, we partially acid-hydrolyzed the CAP by adapting and slightly modifying the method described by Wang et al. (32). The procedure was as follows: 200 mg of CAP was completely dissolved in 20 mL of TFA (0.01 M) and subsequently hydrolyzed at 100 °C for 1 h. After cooling to room temperature, 3 ~ 5 mL of methanol was added, and the residue was then evaporated to dryness using a rotary evaporator (R-300, Buchi, Switzerland). Retention and permeability fractions were collected separately, dialyzed, freeze-dried. Subsequently, the retention fractions were sequentially hydrolyzed with 0.1 M, 0.3 M, and 0.5 M TFA, and treated as above, yielding a total of four retention fractions (0.01-R, 0.1-R, 0.3-R, and 0.5-R) and four permeability fractions (0.01-P, 0.1-P, 0.3-P, and 0.5-P). All the retentate and permeate fractions were acid hydrolyzed and acetylated as described in Section 2.4.1 and analyzed by GC with the same protocol.

#### 2.4.3 Peroxide oxidation and smith degradation

The peroxide oxidation and Smith degradation processes followed previously reported methods with slight modifications (31). Briefly, 20 mg of CAP was dissolved in 20 mL of 15 mM sodium periodate solution and stirred continuously at 4 °C in the dark. Every 12 h, 100 μL of the reaction solution was diluted to 25 mL with distilled water, and the absorbance at 223 nm was measured using a UV spectrophotometer (UV-2550, Shimadzu, Japan) until the absorbance stabilized. Finally, 2 mL of ethylene glycol was added to terminate the reaction. The sodium periodate consumption was calculated using a standard curve (*Y* = 10.3429*X* + 0.0018, *R*^2^ = 0.9997), and the formic acid production was determined by titration with NaOH (0.005 M). After dialyzing the oxidized CAP solution for 24 h, 50 mg of sodium borohydride was added, and the mixture was magnetically stirred at room temperature for 4 h. The pH was then adjusted to neutral with 36% acetic acid, followed by dialysis and freeze-drying to obtain the Smith degradation product. Finally, the degraded components were identified by analyzing the resulting sample using gas chromatography, as described in Section 2.4.1, and comparing them with monosaccharide standards, glycerol (Gly), and erythritol (Ery).

#### 2.4.4 Methylation analysis

Methylation experiments for CAP were performed with reference to previously reported methods and with slight modifications (33). Five milligrams of dry CAP was completely dissolved in 2 mL of anhydrous dimethyl sulfoxide, then 40 mg of sodium hydroxide powder was added, and the reaction was proceeded under N_2_ protection for 4 h. Subsequently, 1 mL of iodomethane was added slowly in an ice-water bath (approximately 40 min), and the reaction was continued under dark conditions for 3 h. The reaction was continued for a further 3 h in an ice-water bath under the protection of N_2_. At the end of the reaction, the extract was extracted with 2 mL of dichloromethane. The extract was dehydrated using a sodium sulfate column (0.5 cm × 15 cm) and hydrolyzed at 100 °C for 6 h. Afterwards, 0.5 mL of sodium boron deuteride (1 M) was added to reduce the reaction over 3 h. The mixture was neutralized with 25% acetic acid, followed by the addition of 2.5 mL of acetic anhydride. After thorough mixing, the reaction was carried out at 100 °C for 2.5 h. The residue was extracted with dichloromethane for five times, and then put into gas phase vials and analyzed using GC–MS (7820A-5975C MSD, Agilent, United States). The GC–MS conditions were as follows: HP-5 MS column (30 m × 0.25 mm × 0.25 μm) at an initial temperature of 120 °C, increased to 210 °C at a rate of 2 °C/min, and held for 2 min. The mass spectrometry results were compared with the PMAA database and related literature, attributed to sugar residues, and the ratio of their relative contents was the ratio of GC peak areas (14).

#### 2.4.5 NMR analysis

The 30 mg of CAP was completely dissolved in 0.5 mL of D_2_O. The solution was centrifuged, and the supernatant was transferred into an NMR tube for analysis. Then, the ^1^H NMR (600 MHz) and ^13^C NMR (150 MHz) spectra were recorded at 25 °C using a High-Resolution NMR spectrometer (Bruker AVANCE AV-600, Rheinstetten, Germany) (18). A standard single-pulse sequence with pre-saturation water suppression (zgpr) was employed for ^1^H NMR, whilst a reverse-gated decoupling pulse sequence (zgig) was used for ^13^C NMR (7). Tetramethylsilane (TMS) was used as the internal standard, with chemical shifts expressed in ppm.

### 2.5 Modification of CAP

The carboxymethylation, acetylation, sulfation and phosphorylation of CAP were carried out according to previous reports with slight modifications (34, 35). Then, the carboxymethylated, acetylated, sulfated and phosphorylated derivatives of CAP, designated CAP-C, CAP-A, CAP-S, and CAP-P, were obtained.

### 2.6 Physical and chemical properties of CAP and its derivatives

#### 2.6.1 Viscosity and solubility analysis

The viscosity ([*η*]) of aqueous solutions of CAP and its derivatives was determined using a Ubbelohde capillary viscometer at 25 ± 0.01 °C (36). All samples were dissolved in aqueous solutions containing NaNO_3_ at concentrations ranging from 0 to 0.1 M. The [*η*] values were determined according to Huggins (2) and Kraemer (3) equations.


$$\frac{\eta_{\mathrm{sp}}}{C}=[\eta ]+K^{\prime }{\left[\eta \right]}^{2}C$$ (2)

$$\frac{\ln\eta_{r}}{C}=[\eta ]-K^{\prime \prime }{\left[\eta \right]}^{2}C$$ (3)

Where *η_r_* is the viscosity, *η_sp_* is the specific viscosity, *C* is the polysaccharide concentration (mg/dL), *K′* is characteristic of a given solute-solvent system, and *K″* is a constant dependent on the polymer and solvent. The linear functions were extrapolated to the concentration of zero to obtain the [*η*] value at the intercept.

The solubility determination of CAP and its derivatives was based on the method of Wang et al. (37). 1.0 g of the polysaccharide sample was dissolved in 100 mL of distilled water with continuous stirring at 500 rpm/min at room temperature. After centrifugation at a specific time point, the undissolved polysaccharide fraction was collected, freeze-dried and placed in a vacuum oven at 50 °C until constant weight. The solubilities were calculated according to Equation 4.


$$\text{Solubility}\;(\%)=\frac{\left(W_{s}-W_{i}\right)}{W_{i}}\times 100\%$$ (4)

Where *W_i_* is the initial weight of the sample (g) and *Ws* is the weight of dried insoluble polysaccharide (g).

#### 2.6.2 Components determination

The total sugar content of CAP and its derivatives was determined by the phenol-sulfuric acid method (33). Five milligram sample of polysaccharide was mixed with 100 mL of 5% phenol solution, followed by the addition of 1 mL of concentrated sulfuric acid. The mixture was reacted at 90 °C for 10 min. Then, the absorbance value at 490 nm was used to calculate the total sugar content by glucose standard curve.

The carbazole-sulfuric acid method was used to determine the uronic acid content of CAP and its derivatives (38). Five milligram of polysaccharide sample was mixed with 2 mL of concentrated sulfuric acid and heated at 90 °C for 30 min. After cooling, 100 μL of carbazole solution (0.1% in anhydrous ethanol) was added, and the mixture was allowed to stand for 90 min. Then, the absorbance value was measured at 530 nm, and the glucuronic acid content was calculated by uronic acid standard curve.

The protein content of CAP and its derivatives was determined using the Coomassie Brilliant Blue method (39). Specifically, 1 mL of polysaccharide solution was mixed with 5 mL of Coomassie Brilliant Blue G-250, and the reaction was carried out at room temperature for 10 min. Then, the absorbance value was measured at 595 nm, and the protein content was calculated by using the standard curve of bovine serum albumin (BSA).

#### 2.6.3 Molecular weight determination

The weight average molecular weight of CAP and its derivatives was determined by HPSEC (28). The polysaccharide samples were analyzed on an Agilent 1260A instrument equipped with a TSK-GEL G4000 PWXL column (7.8 mm × 300 mm) and a RID−10A differential detector under the following chromatographic conditions: distilled water as the mobile phase at a flow rate of 0.6 mL/min, column temperature of 35 °C, and sample volume of 35 μL. The standard dextran was prepared in double-distilled water at a concentration of 0.2%.

#### 2.6.4 UV and FT-IR determination

The UV–visible spectrophotometer (UV2550, Shimadzu, Japan) was used to determine the UV absorption spectra of CAP and its derivatives in the range of 200 ~ 400 nm (40). In this experiment, the sample was a 1 mg/mL aqueous solution, and distilled water was used as a blank control.

The FT-IR of CAP and its derivatives in the wavelength range of 4,000 ~ 500 cm^−1^ were determined by KBr pressing method using a Fourier Transform Infrared Spectrometer (IR Tracer−100, Shimadzu, Japan) (41).

#### 2.6.5 Congo red determination

The Congo red assay was performed as previously reported with minor modifications (42). Briefly, 1 mL of a 3.0 mg/mL aqueous solution of the sample was mixed with 1 mL of 0.2 mM Congo red solution, and the reaction was kept away from light for 30 min, then 3 mL of NaOH solution with different concentrations (0, 0.1, 0.2, 0.3, 0.4, and 0.5 M) were added. The absorption spectra were scanned in the wavelength range of 400 ~ 600 nm using a UV–visible spectrophotometer (UV2550, Shimadzu, Japan), and the shift change of the maximum absorption wavelength (λ_max_) was recorded.

#### 2.6.6 SEM determination

The microscopic morphology of CAP and its derivatives was observed using a scanning electron microscope (7,500 F, Jeol, Japan) (35). In this experiment, the samples were uniformly dispersed on conductive adhesive tape, and following gold plating treatment (thickness of about 10 nm), the observations were conducted under an accelerating voltage of 10 kV and a working distance of 8 mm.

#### 2.6.7 XRD determination

The crystallographic characterization of CAP and its derivatives was performed using an X-ray diffractometer (D8 Advance, Bruker, Germany) (31, 40). Appropriate amounts of polysaccharide samples were scanned in the range of 5 ~ 80° at room temperature. The scanning parameters were set as follows: a step size of 0.01° and a scanning speed of 0.1 s/step.

#### 2.6.8 Thermal analysis

The thermal analysis of CAP and its derivatives was conducted using thermogravimetric analysis (TG), differential thermogravimetric analysis (DTG), and differential scanning calorimetry (DSC) with a simultaneous thermal analyzer (STA200, Jeol, Japan) (42). In this experiment, 5 mg of the sample was placed in a platinum crucible and heated from 25 °C to 600 °C at 10 °C/min under argon protection.

### 2.7 Anti-inflammatory activity determination

#### 2.7.1 Cell culture and establishment of the RAW264.7 macrophages inflammation model

The RAW264.7 macrophage cell line was cultured in RPMI 1640 medium supplemented with 10% fetal bovine serum (FBS) and antibiotics (penicillin: 100 U/mL, streptomycin: 100 μg/mL), and maintained in an incubator at 37 °C with 5% CO₂. The culture medium was replaced every 48 h.

Cells in the logarithmic growth phase were seeded in 96-well plates at a density of 1 × 10^4^ cells/mL (100 μL per well) and cultured for 12 h. The medium was then replaced with serum-free RPMI 1640, followed by incubation for 2 h. Subsequently, 2 μg/mL lipopolysaccharide (LPS) was added to induce an inflammatory response for 24 h, thereby establishing the RAW264.7 macrophage inflammation model (43).

#### 2.7.2 RAW264.7 macrophage viability determination

The cell suspension (1 × 10^4^ cells/mL) was added to a 96-well plate in a volume of 100 μL. After 12 h of incubation, all cells were divided into a blank group, control group, and sample groups. The sample groups were treated with different concentrations (10, 20, 60, 100, and 200 μg/mL) of CAP and its derivatives, respectively. Normal RAW264.7 macrophages cultured solely with medium throughout the process served as the normal group, while cells cultured with 2 μg/mL LPS were used as the model group. After 24 h of treatment for each group, the culture medium was removed following centrifugation, and 20 μL of 5 mg/mL MTT solution and 180 μL of fresh medium were added to each well (26). Absorbance was measured at 490 nm after 4 h. Cell viability was calculated according to Equation 5.

(5) $$\text{Cell viability}\;(\%)=\frac{\left(A_{s}-A_{b}\right)}{\left(A_{c}-A_{b}\right)}\times 100\%$$

Where *A_s_*, *A_c_*, and *A_b_* are the absorbance values of the sample, model and normal groups, respectively.

#### 2.7.3 NO, PGE2 and proinflammatory cytokines determination

The NO content in the culture medium supernatant was measured using the nitrate reductase method provided in the NO detection kit (44). The levels of PGE_2_, IL-6, IL-1β, and TNF-α in the culture medium supernatant were determined using the ELISA method according to their respective detection kits (IL-6, IL-1β, TNF-α, and PGE_2_), with all procedures performed in accordance with the manufacturer’s instructions (41, 43).

### 2.8 Statistical analysis

All experiments were independently repeated three times (*n* = 3). The experimental data are expressed as mean ± standard deviation (mean ± SD). Single-way analysis of variance (ANOVA) was used to test the significance of differences between groups. For indicators showing statistical significance (*p* < 0.05) in the ANOVA analysis, Duncan’s multiple comparison test was further employed for *post hoc* analysis. Graphs related to anti-inflammatory activity were generated using GraphPad Prism 10.0 software, NMR spectral data were processed using MestReNova software, and all other charts were created using Origin 2024 software.

## 3 Results and discussions

### 3.1 Single factor optimization

**Figure 1:**
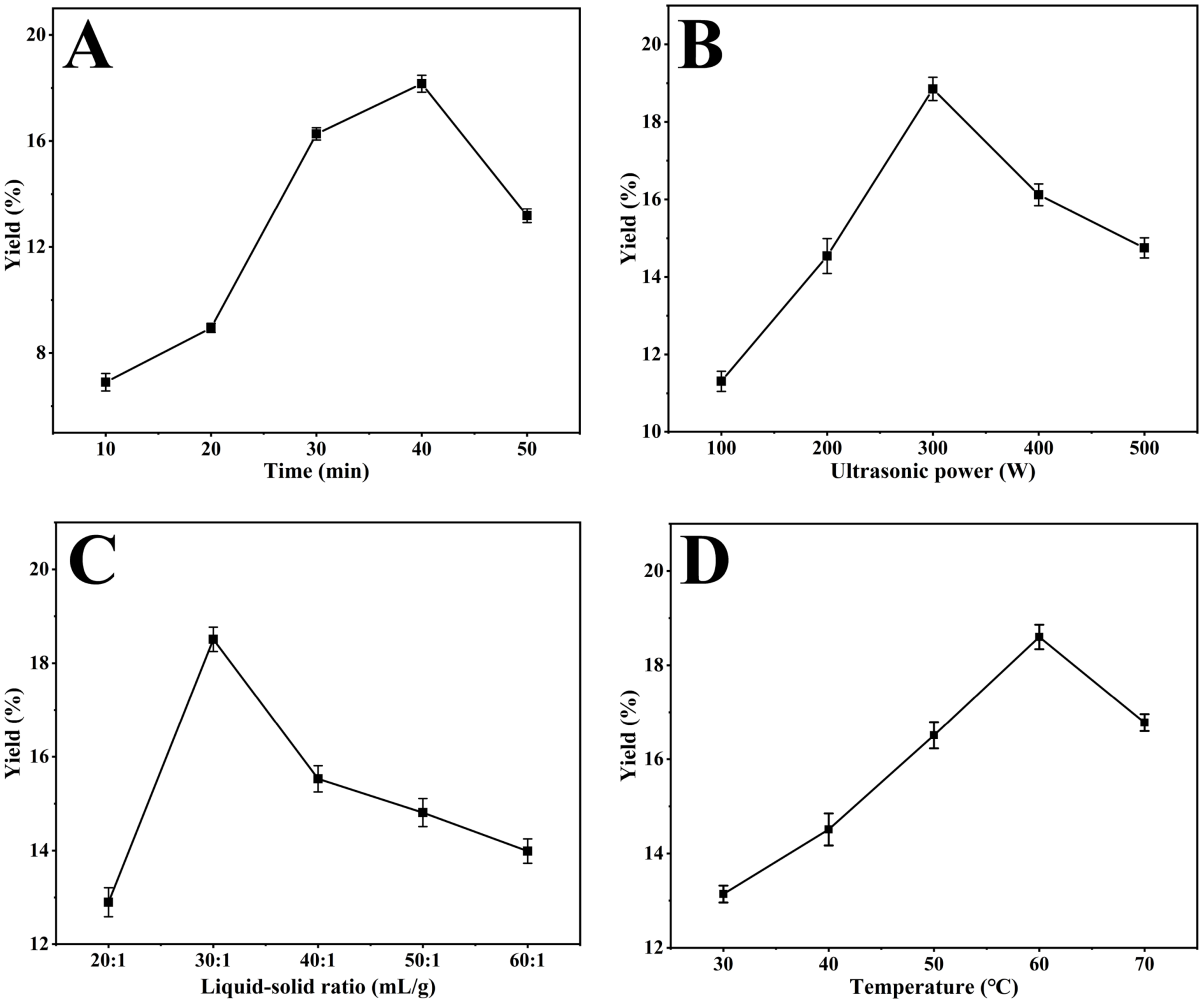
Effect of variables on crude polysaccharide yield. **(A)** Time (min), **(B)** ultrasonic power (W), **(C)** liquid–solid ratio (mL/g), **(D)** temperature (°C).

The polysaccharide extraction process exhibits time dependency. Insufficient extraction time prevents complete extraction of polysaccharides, and conversely, prolonged extraction time leads to dissolution of more impurities, which reduces the yield of polysaccharides (45). As shown in Figure 1A, the crude polysaccharide yield increased significantly with the extension of extraction time, reaching a peak of 18.16 ± 0.32% at 40 min, and then decreased gradually with the extension of extraction time.

In the process of polysaccharide leaching, ultrasound induces dynamic deformation of the cell membrane through its special physical effects, thereby promoting the release of intracellular substances (46). Several studies have shown that ultrasonic power directly affects polysaccharide yield (47–49). As shown in Figure 1B, the yield of crude polysaccharides increased significantly with increasing ultrasonic power, reaching a peak of 18.85 ± 0.30% at 300 W. However, the yield started to decrease when the ultrasonic power exceeded 300 W.

A suitable liquid–solid ratio is crucial in the extraction of plant polysaccharides. The optimum liquid–solid ratio helps to create an osmotic pressure at the plant cell membrane, thus promoting polysaccharide release (50). However, when the liquid–solid ratio continued to increase, the excess solvent would weaken the ultrasonic energy, leading to yield decrease (51). As shown in Figure 1C, the crude polysaccharide yield increased with an increasing liquid–solid ratio, and the yield reached a peak of 18.51 ± 0.26% at 20:1 mL/g. If the liquid–solid ratio continued to increase, the yield decreased.

Temperature control is a key factor in the extraction of plant polysaccharides. If the temperature is not high enough, the polysaccharides may not be fully released (48). On the contrary, when the temperature is too high, it leads to polysaccharide degradation or impurity leaching, which reduces polysaccharide yield (40). As shown in Figure 1D, the crude polysaccharide yield peaked at 18.60 ± 0.26% when the extraction temperature was 60 °C. As the temperature continues to rise, the yield begins to decline.

### 3.2 RSM optimization and model validation

The effects of time (A), ultrasonic power (B), liquid–solid ratio (C) and temperature (D) on crude polysaccharide yield were investigated using BBD, and the results are shown in Table 1. The second-order polynomial equation established by multiple regression analysis is as follows:


$$\begin{array}{l} Y=18.82–0.08\;A–0.71\;B+0.47\;C+0.08\;D–0.34\;\mathrm{AB}\\ –0.26\;\mathrm{AC}–0.07\;\mathrm{AD}+0.52\;\mathrm{BC}+0.07\;\mathrm{BD}–0.06\;\mathrm{CD}\\ –2.04\;A^{2}–1.73\;B^{2}–1.41\;C^{2}–1.21\;D^{2}. \end{array}$$

The results of ANOVA and the significance test of the BBD model are presented in Table 2. Its *F-*value is 13.94, *p* < 0.0001, indicating that the model is highly consistent with the experimental results and statistically significant (28). The *F*-value of the lack of fit is 4.20, with a *p*-value of 0.06. And the coefficient of determination, *R*^2^ = 0.9458, shows that the experimental results are in good agreement with the predicted values of the polynomial model (46). The adjusted coefficient of determination, *R*^2^*_adj_* = 0.9315, confirms that the model has good predictive accuracy and generalizability (49). The low coefficient of variation (*CV* = 2.80%) indicates that the experimental data have high accuracy and reliability (52). In addition, the order of influence of each factor was: liquid–solid ratio (C) > ultrasonic power (B) > time (A) > temperature (D).

**Table 2:** The ANOVA of response surface quadratic model for crude polysaccharide yield.

Source	Sum of squares	Df	Mean square	*F*-value	*P*-value	Significance
Model	0.3367	1	0.3367	13.94	<0.0001	**
*A*	4.66	1	4.66	0.99	<0.0001	**
*B*	4.80	1	4.80	3.83	0.0006	**
*C*	0.0833	1	0.0833	7.55	<0.0001	**
*D*	0.0289	1	0.0289	0.20	0.1742	
*AB*	0.2601	1	0.2601	0.26	0.6202	
*AC*	0.0210	1	0.0210	2.19	0.1613	
*AD*	0.0012	1	0.0012	0.68	0.7641	
*BC*	0.0210	1	0.0210	0.094	0.9476	
*BD*	0.0169	1	0.0169	1.24	0.2834	
*CD*	22.83	1	22.83	0.085	0.9277	
*A* ^2^	21.42	1	21.42	171.69	<0.0001	**
*B* ^2^	16.94	1	16.94	0.21	0.0651	
*C* ^2^	8.84	1	8.84	0.78	0.0393	*
*D* ^2^	3.22	14	0.2299	0.93	0.3514	
Residual	3.22	10	0.3216			
Lack of fit	0.0023	4	0.0006	4.20	0.06	
Pure error	59.33	28				
Cor total	0.3367	1	0.3367			

*R*^2^ = 0.9458; *R*^2^_adj_ = 0.9315; *C. V.%* = 2.80; *P < 0.05; **P < 0.01.

**Figure 2:**
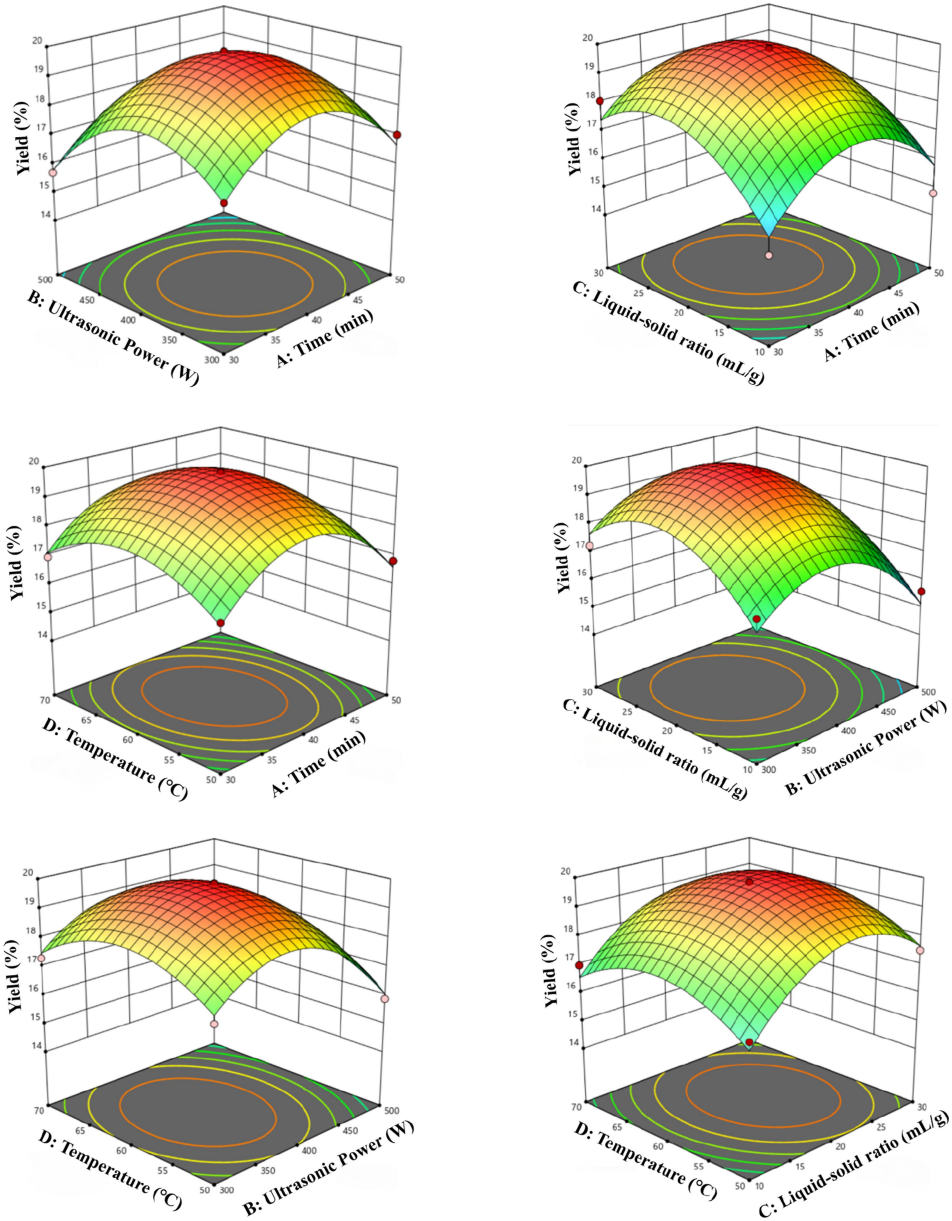
Response surface plots of factor interactions.

In order to study the interaction between the variables and to determine the optimal conditions for maximum yield, 3D response surface plots (Figure 2) and 2D contour plots (Supplementary Figure S1) were plotted. As the response surface curves become more elliptical, the interaction between variables becomes stronger (53). The optimal extraction conditions predicted by the secondary model were as follows: a time of 42.61 min, an ultrasonic power of 308.14 W, a liquid–solid ratio of 20.68:1 mL/g, and a temperature of 61.85 °C. The theoretical yield of crude polysaccharides reached 19.23 ± 0.08%. Based on the practical feasibility, the parameters were adjusted to a time of 43 min, an ultrasonic power of 310 W, a liquid–solid ratio of 21:1 mL/g, and a temperature of 62 °C. Three validation experiments were conducted under these optimized conditions, and the average crude polysaccharide yield was measured to be 19.09 ± 0.12%.

### 3.3 Isolation and purification

**Figure 3:**
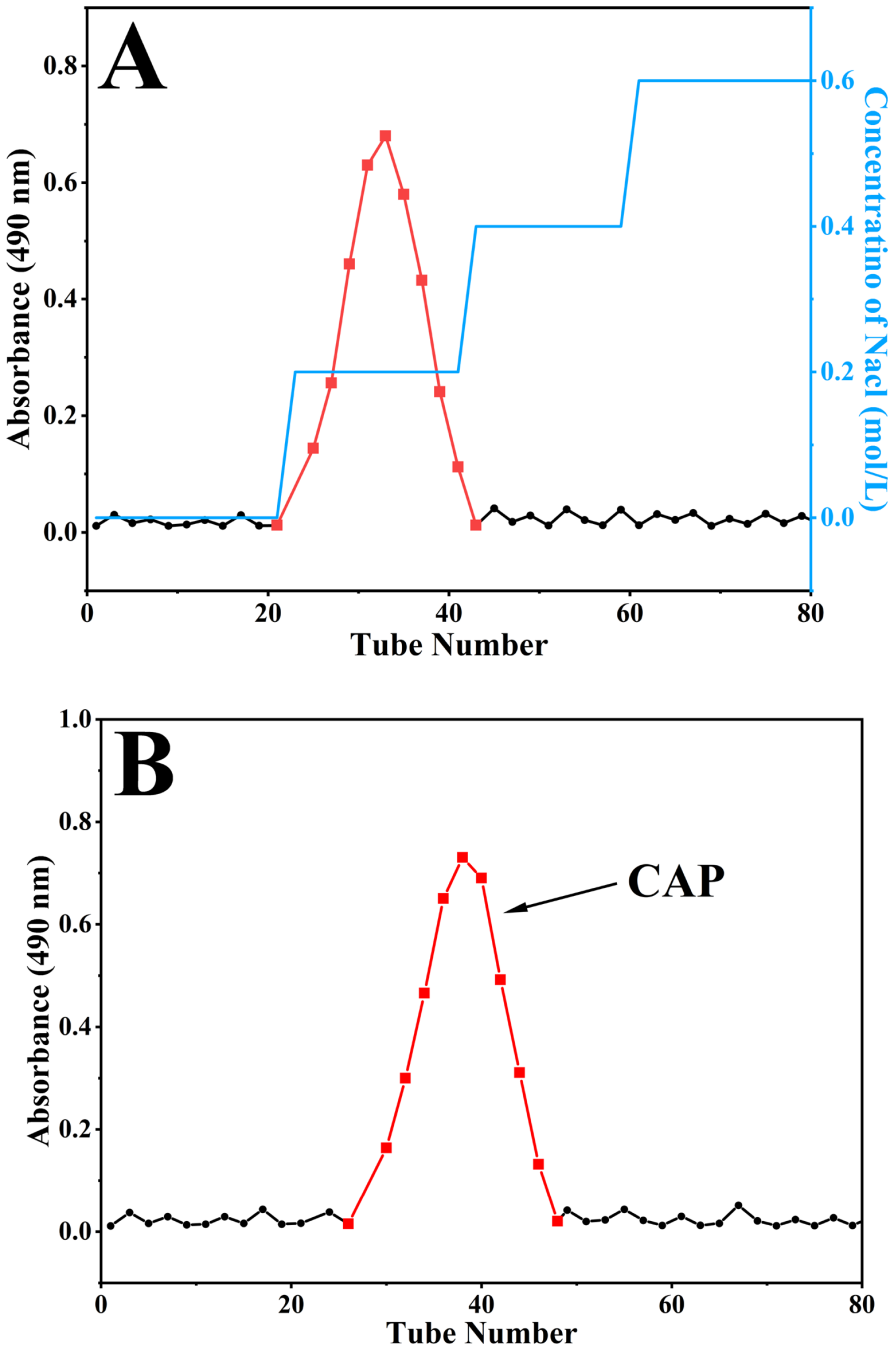
Purification of CAP. DEAE-650 M column elution curve **(A)**; Sephadex G-75 column elution curve **(B)**.

Based on the elution curve of the DEAE-650 M column, a major eluting fraction was obtained from 0.2 M NaCl (Figure 3A), yielding a recovery rate of 48.74%. This fraction was collected and further purified using a Sephadex G-75 column to obtain a single elution peak (Figure 3B). This peak was then collected, dialyzed, and freeze-dried to produce a high-purity polysaccharide fraction (CAP) with a yield of 29.8%.

### 3.4 Structural characterization of CAP

#### 3.4.1 Partial acid hydrolysis

The differential degradation of monosaccharides during acid hydrolysis can reveal their structural localization within polysaccharides, providing an important basis for resolving their molecular architectures. Stepwise acid hydrolysis is typically employed to separately analyze the retention and permeate parts via gas chromatography. The results of partial acid hydrolysis of CAP (Table 3; Supplementary Figure S2) revealed significant differences (*p* < 0.05) in monosaccharide composition at different TFA concentrations. When the TFA concentration was increased from 0.01 M to 0.1 M, the Rha (from 25.38 to 35.46%) and Gal (from 16.78 to 32.41%) contents in the retention part increased dramatically, whereas the Ara content (from 34.66 to 3.51%) decreased significantly (*p* < 0.05), and the remaining monosaccharides demonstrated only a minor increase. In contrast, the permeate part exhibited a significant increase in Ara content (from 16.56 to 22.38%), while the rest of the monosaccharides did not change significantly.

**Table 3:** The results of partial acid hydrolysis of CAP.

Sample	Molar ratio (%)					
	Rha	Ara	Xyl	Man	Glc	Gal
Retentate part						
0.01-R	25.38	34.66	12.13	1.73	9.32	16.78
0.1-R	36.46	3.51	13.57	2.69	11.36	32.41
0.3-R	25.15	9.05	5.39	3.73	12.11	34.57
0.5-R	9.41	1.11	18.64	7.25	15.27	48.32
Permeate part						
0.01-P	24.32	16.56	11.28	7.60	10.97	29.27
0.1-P	26.54	22.38	10.07	6.14	8.44	26.43
0.3-P	13.62	34.31	28.22	3.35	5.61	14.79
0.5-P	27.75	53.61	9.47	0.98	1.37	6.82

0.01-R, 0.1-R, 0.3-R, and 0.5-R indicating the retentate part of the 0.01, 0.1, 0.3, and 0.5 M TFA hydrolyzed CAP. 0.01-P, 0.1-P, 0.3-P, and 0.5-P indicating the permeate part of the 0.01, 0.1, 0.3, and 0.5 M TFA hydrolyzed CAP.

In addition, the monosaccharide content of each fraction showed irregular changes with increasing TFA concentration. Notably, in 0.5 M retention part (0.5-R), the contents of Xyl (18.64%), Man (7.25%), Glc (15.27%), and Gal (48.32%) peaked, while those of Rha (9.41%) and Ara (1.11%) decreased to the minimum. In contrast, in 0.5 M permeate part (0.5-P), the contents of Rha (27.75%) and Ara (53.61%) were the highest, while those of Xyl (9.47%), Man (0.98%), Glc (1.37%), and Gal (6.82%) were significantly lower (*p* < 0.05). These results indicate that Rha and Ara are predominantly located at the terminal positions of CAP side chains, while Xyl may be distributed between both the main chain and the terminal positions of the side chains. In contrast, Man, Glc, and Gal likely form the core structural framework of CAP, constituting its backbone. This finding is consistent with the results reported by Lin et al. (54), whose study on *Lonicera japonica* polysaccharide acid hydrolysis demonstrated that Ara was the most abundant monosaccharide (65.89%) and was primarily localized in the polysaccharide side chains, whereas galacturonic acid (GalA), the least abundant component, likely constituted the backbone structure.

#### 3.4.2 Periodate oxidation and smith degradation

Smith degradation involves periodate oxidation of polysaccharides followed by reduction to produce stable polyols. These polyols are then acetylated and analyzed by gas chromatography to obtain unoxidized monosaccharides and degradation products (Gly and Ery) (55). The glycosidic linkages of CAP were resolved by periodate oxidation combined with Smith degradation, including reduction, acetylation and GC analysis. As shown in Table 4, CAP consumes 0.82 mmol of periodate and produces 0.08 mmol of HCOOH during oxidation, with a ratio of 10.25:1, suggesting the presence of a 1 → or 1 → 6 linkage or trace branching structure (31). In addition, the ratio of periodate consumption to HCOOH production was significantly greater than 2, suggesting that 1 → 2, 1 → 2,6, 1 → 4, or 1 → 4,6 glycosidic bonding may exist in the polysaccharides (56). Notably, the incomplete consumption of periodate indicates that CAP may contain glycosidic linkages that cannot be oxidized, such as 1 → 3, 1 → 3,6, 1 → 2,3, 1 → 2,4, or 1 → 3,4 types. This observation aligns with the glycosidic bonding patterns reported by Albano et al. (57) in their study of *Styela plicata* polysaccharides.

**Table 4:** The results of periodate oxidation and Smith degradation of CAP.

Samples	CAP
Periodate oxidation (mmol)	
Consumption of NaIO_4_	0.82
Production of HCOOH	0.08
Smith degradation (%)	
Gly	20.45
Ery	12.79
Rha	10.74
Ara	12.46
Xyl	10.67
Man	4.94
Glc	3.41
Gal	24.54

The structure of CAP was further elucidated by Smith degradation. GC analysis of the degradation products (Table 4; Supplementary Figure S3) detected Rha, Ara, Xyl, Man, Glc, and Gal, suggesting the presence of unoxidized glycosidic linkages such as 1 → 3, 1 → 3,4, or 1 → 2,4 (54). Notably, Gal accounted for 24.54% of the composition, confirming its role as a major constituent of the CAP backbone. This finding aligns with the partial acid hydrolysis results presented in Section 3.4.1. The presence of Gly (20.45%) in the degradation products indicated that the polysaccharides were enriched in 1 → 2, 1 → 2,6, or 1 → 6 linkages, whereas the presence of Ery (12.79%) suggested 1 → 4 or 1 → 4,6 linkages (58). In addition, the molar ratio of Gly, Ery, Rha, Ara, Xyl, Man, Glc, and Gal was 6: 3.75: 3.15: 3.65: 3.13: 1.45: 1: 7.20. The results indicate that the CAP backbone may contain various glycosidic linkages, including 1 → 2, 1 → 2,4, 1 → 3, 1 → 3,6, 1 → 4, or 1 → 6 types. This linkage pattern shows similarities to those reported by Liu et al. (59) for *Craterellus cornucopioides* polysaccharides (1 → 3, 1 → 6, 1 → 2, 1 → 4, or 1 → 3,6 linkages).

#### 3.4.3 Methylation analysis

Methylation analysis is an important tool for resolving polysaccharide glycosidic bonding patterns and complex chemical structures. The results of the methylation analysis (Table 5) showed that CAP exhibits complex glycosidic bond types containing four main monosaccharide residues: Xyl, Man, Glc and Gal. Among them, Gal residues accounted for the highest proportion, comprising →3)-Gal*p*-(1 → (13.59%), Gal*p*-(1 → (16.71%), and →2,4)-Gal*p*-(1 → (11.43%). Xyl residues were the next most abundant, with →3)-Xyl*p*-(1 → (8.61%) and →4)-Xyl*p*-(1 → (4.91%). Glc residues were primarily Glc*p*-(1 → (6.84%) and →6)-Glc*p*-(1 → (2.27%), while Man residues included Man*p*-(1 → (5.89%) and →3,6)-Man*p*-(1 → (2.86%). Notably, CAP contained higher molar percentages of terminal sugar residues, including T-Rha*p* (9.74%), T-Xyl*p* (10.72%), and T-Ara*f* (6.43%). Further analysis demonstrated that the 12 structural units T-Rha*p*, T-Ara*f*, →3)-Xyl*p*-(1→, T-Xyl*p*, →4)-Xyl*p*-(1→, Man*p*-(1→, →3,6)-Man*p*-(1→, Glc*p*-(1→, →6)-Glc*p*-(1→, →3)-Gal*p*-(1→, Gal*p*-(1→, and →2,4)-Gal*p*-(1 → have a molar ratio of 4.29: 2.83: 3.79: 4.72: 2.16: 5.59: 1.26: 3.01: 1.00: 5.99: 7.36: 5.04. These data demonstrated that Gal residues (→3)-Gal*p*-(1→, Gal*p*-(1→, and →2,4)-Gal*p*-(1→) constitute the highest proportion (41.73%), thereby confirming that the CAP backbone structure is primarily composed of Gal units. Moreover, the presence of terminal residues (T-Rha*p* (9.74%), T-Ara*f* (6.43%), and T-Xyl*p* (10.72%) indicates that the polysaccharide possesses a long, straight-chain structure with low branching (58). This conclusion is consistent with the results of partial acid hydrolysis and Smith degradation analyses, which together reveal the basic structural features of CAP.

**Table 5:** The results of methylation analysis for CAP.

Sugar residues	Methylated sugars	Linkage patterns	Relative amount (mol %)
Rha	3,4-Me_2_-Rha	T-Rha*p*	9.74
Ara	2,4,5-Me_3_-Ara	T-Ara*f*	6.43
Xyl	2,5-Me_2_-Xyl	→3)-Xyl*p*-(1→	8.61
	2,3,4-Me_3_-Xyl	T-Xyl*p*	10.72
	2,3-Me_2_-Xyl	→4)-Xyl*p*-(1→	4.91
Man	2,3,4,6-Me_4_-Man	Man*p*-(1→	5.89
	2,4-Me_2_-Man	→3,6)-Man*p*-(1→	2.86
Glc	2,3,4,6-Me_4_-Glc	Glc*p*-(1→	6.84
	2,3,4-Me_3_-Glc	→6)-Glc*p*-(1→	2.27
Gal	2,4,6-Me_3_-Gal	→3)-Gal*p*-(1→	13.59
	2,3,4,6-Me_4_-Gal	Gal*p*-(1→	16.71
	3,6-Me_2_-Gal	→2,4)-Gal*p*-(1→	11.43

#### 3.4.4 NMR analysis

**Figure 4:**
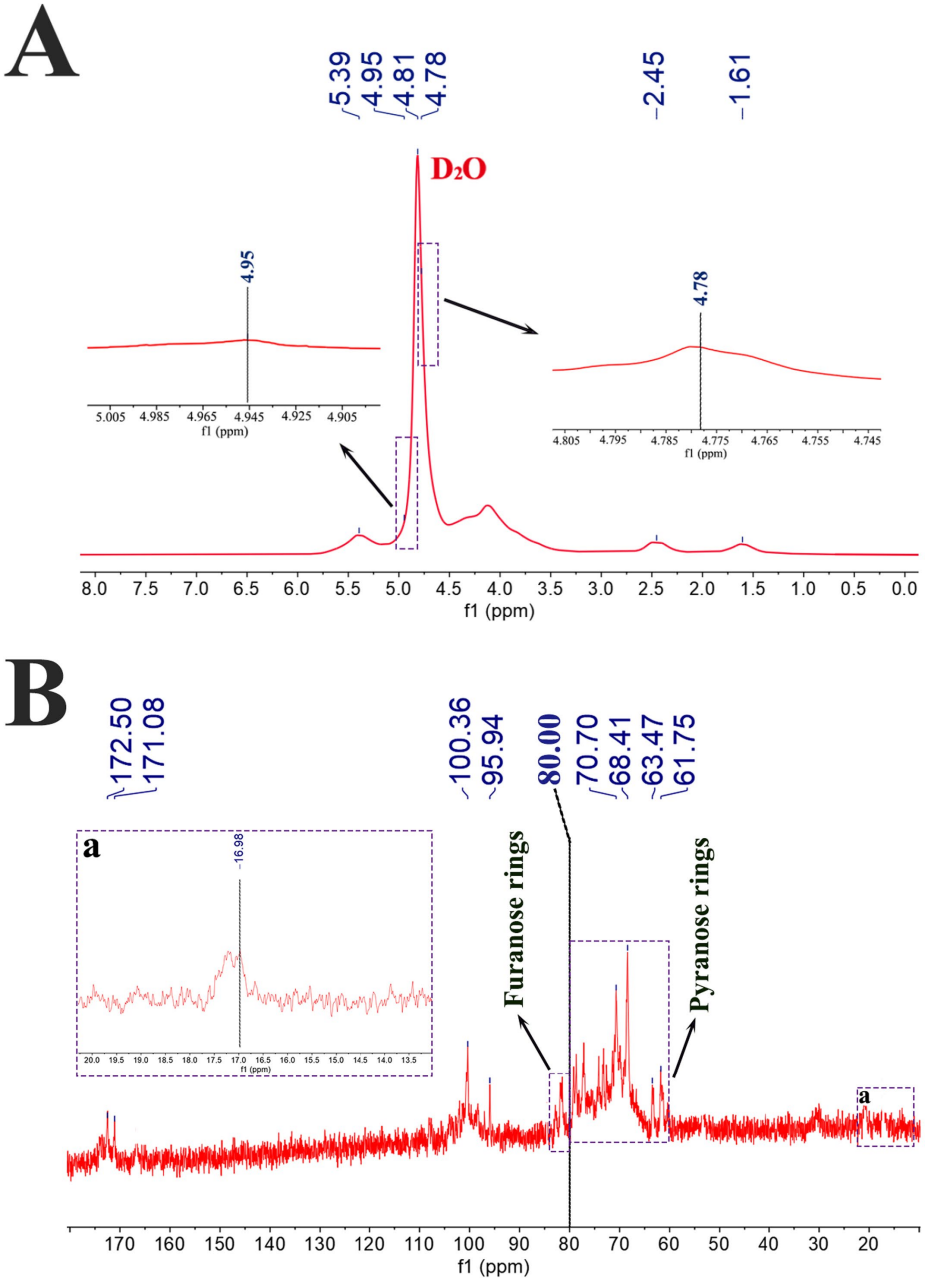
^1^H NMR **(A)** and ^13^C NMR **(B)** spectra of CAP.

NMR is the most powerful tool for determining polysaccharide structures. In the ^1^H NMR spectrum of CAP (Figure 4A), the signal at *δ* 4.81 ppm corresponds to the D₂O solvent, while the signals at *δ* 4.78 ppm, δ 4.95 ppm and δ 5.39 ppm are assigned to the anomeric protons (50). Among these, the signals at δ 4.95 ppm and *δ* 5.39 ppm indicate the presence of α-glycosidic bonds in CAP (40). In addition, the characteristic peaks at *δ* 1.61 ppm and δ 2.45 ppm were assigned to the methyl groups of Rha residues and the methyl group of an N/O-acetyl group, respectively (60). The ^13^C NMR spectrum (Figure 4B) revealed two signals (*δ* 95.94 ppm and *δ* 100.36 ppm) within the typical anomeric carbon region (*δ* 90 ~ 110 ppm), confirming the presence of two distinct sugar units (61). The multiple signal peaks in the range of *δ* 65 ~ 85 ppm are assigned to the C2-C5 resonances of the polysaccharide (17). The peaks at δ 61.75 ppm and δ 63.47 ppm are attributed to C6 resonance signals (62). The signal at δ 16.98 ppm (a) originates from the methyl carbon (C6) of Rha, consistent with characteristic peaks in the ^1^H NMR spectrum (δ 1.61 ppm and δ 2.45 ppm) (50). Furthermore, the chemical shifts of C3-C5 in furanose rings typically exceed δ 80 ppm, whereas those in pyranose rings appear below this value (21). The chemical shift distribution of C2-C6 in CAP was distributed on both sides of δ 80 ppm, indicating the presence of both pyranose and furanose ring structures, with the pyranose ring predominating. Two characteristic signals at δ 171.08 ppm and δ 172.50 ppm (within the δ 170 ~ 180 ppm range) were assigned to substituted and non-substituted α-D-galacturonic acid (α-D-Gal*p*A) residues, respectively, indicating the presence of trace uronic acid components in CAP (63). Integrated analysis of the ^1^H and ^13^C NMR spectra revealed that the ^1^H signal at δ 5.39 ppm, coupled with the ^13^C signals at δ 68.41 ppm and δ 70.70 ppm, indicates the presence of an α-(1 → 6)-linked glycosidic chain in CAP (28). Meanwhile, the ^1^H signal at δ 4.78 ppm corresponds to a 1,3-linked galactose-pyranose (1,3-Gal*p*) structural unit (64).

### 3.5 Physical and chemical properties of CAP and its derivatives analysis

#### 3.5.1 Physicochemical properties and composition analysis

Table 6 presents the viscosity, solubility, total sugar content, protein content, uronic acid content, Mw (Supplementary Figure S4), degree of substitution (DS) and monosaccharide composition (Supplementary Figure S5) of CAP and its derivatives. After different chemical modifications, the physicochemical properties of CAP and its derivatives exhibited variations. Viscosity and solubility can directly reflect the hydrodynamic properties and hydrophilicity of polysaccharides (65). The measurement results demonstrate that chemical modifications significantly alter the viscosity and solubility of polysaccharides, and these changes are closely related to the characteristics of substituent groups and modifications in molecular conformation (66). Among them, compared to the natural polysaccharide (CAP), both CAP-C and CAP-P exhibited significantly higher viscosity (10.82 ± 0.09 dL/g and 10.21 ± 0.11 dL/g, respectively) and solubility (98.84 ± 0.24% and 91.27 ± 0.38%, respectively). This enhancement can be attributed to the introduced groups’ strong ionization capacity and hydrophilic properties, which not only promote the extension of polysaccharide molecular chains, but also simultaneously enhance their hydration (67). In contrast, CAP-A exhibited significantly lower viscosity (8.22 ± 0.08 dL/g) and solubility (64.17 ± 0.33%) (*p* < 0.05), which may be attributed to the hydrophobicity of the acetyl group, causing the curling of polysaccharide molecular chains and a reduction in hydration capacity (68). Notably, CAP-S exhibited lower viscosity (8.92 ± 0.14 dL/g) than the natural polysaccharide, while its solubility (93.94 ± 0.51%) was significantly increased (*p* < 0.05). This may be attributed to the bulky sulfate group altering the molecular chain conformation, resulting in enhanced backbone rigidity and reduced viscosity (37). However, the electrostatic repulsion induced by its negative charge prevented the aggregation of polysaccharide chains, overcoming intermolecular forces and thereby enhancing the hydration capacity of the polysaccharide (36). Yu’s team (61) successfully reduced the polysaccharide viscosity by performing sulfation modification of jackfruit polysaccharide. On the other hand, Liu’s team (69) significantly improved the solubility of *Atractylodes lancea* polysaccharide by sulfation modification. These successful cases collectively demonstrate that the physicochemical properties of polysaccharides can be modulated through chemical modifications.

**Table 6:** Viscosity, solubility, and chemical composition analysis of CAP and its derivatives.

Samples	CAP	CAP-C	CAP-A	CAP-S	CAP-P
Viscosity (dL/g)	9.78 ± 0.04^a^	10.82 ± 0.09^b^	8.22 ± 0.08^b^	8.92 ± 0.14^b^	10.21 ± 0.11^a^
Solubility (%)	86.76 ± 0.46^a^	98.84 ± 0.24^b^	64.17 ± 0.33^b^	93.94 ± 0.51^b^	91.27 ± 0.38^b^
Total sugar (%)	84.34 ± 0.48^a^	40.27 ± 0.31^d^	52.74 ± 0.39^c^	59.63 ± 0.41^c^	71.22 ± 0.45^b^
Protein (%)	1.06 ± 0.05^a^	1.13 ± 0.02^a^	0.96 ± 0.08^a^	0.92 ± 0.02^a^	0.98 ± 0.04^a^
Uronic acid (%)	8.43 ± 0.13^a^	5.18 ± 0.16^c^	5.97 ± 0.12^c^	7.45 ± 0.14^b^	3.14 ± 0.08^d^
Mw (kDa)	286.49^a^	82.38^b^	269.17^a^	274.85^a^	271.35^a^
DS or content (wt %)	/	0.675	0.426	0.232	0.384
Monosaccharide composition (mol-%)					
Rha (%)	23.22	29.59	22.99	23.19	15.06
Ara (%)	18.35	19.23	18.11	18.84	19.02
Xyl (%)	18.73	8.88	12.64	13.05	10.62
Man (%)	5.24	5.62	4.89	4.35	4.75
Glc (%)	3.74	14.79	9.76	10.14	10.31
Gal (%)	30.72	21.89	31.61	30.43	40.24

“/” represents not detected. Data are presented as mean ± standard deviation (SD) (*n* = 3). Values not marked with the same superscript letter indicate significant differences according to Duncan’s multiple range test (*P* < 0.05).

Chemical composition analysis indicated that the total sugar content of all derivatives was significantly lower than that of CAP (*p* < 0.05), in the following order: CAP (84.34 ± 0.48%) > CAP-P (71.22 ± 0.45%) > CAP-S (59.63 ± 0.41%) > CAP-A (52.74 ± 0.39%) > CAP-C (40.27 ± 0.31%). This phenomenon was mainly due to the degradation of polysaccharide molecular chains triggered by the elimination reaction during the modification process (70). In addition, the protein content of all derivatives did not change significantly (*p* > 0.05), whereas the uronic acid content exhibited a difference significantly (*p* < 0.05), which may be attributed to varying modification conditions affecting neutral sugar conversion and uronic acid degradation (71). Similar phenomena have been previously reported by Nuerxiati et al. (34).

Mw analysis showed that the Mw of all derivatives was lower than that of CAP (286.49 kDa). Among them, CAP-C (82.38 kDa) exhibited the most significant decrease (*p* < 0.05), which could be attributed to glycosidic bond breakage triggered by the alkaline and high-temperature modification conditions (72). The relatively small changes in the Mw of the other derivatives suggest that their structures remain largely intact, which is consistent with the findings of Xie et al. (73) in their study on *Inonotus obliquus* polysaccharides. In addition, the substitution degrees of CAP-C, CAP-A, CAP-S, and CAP-P were 0.675, 0.426, 0.232, and 0.384, respectively. These values were consistent with the substitution degrees reported by Yang and Huang (27) for *Solanum tuberosum* polysaccharides (CM-STP: 0.699, AC-STP: 0.409, S-STP: 0.211, and P-STP: 0.387).

Monosaccharide composition analysis revealed that CAP and its derivatives contained the same types of monosaccharide fractions, but the relative content of each monosaccharide was significantly different (*p* < 0.05). Among them, Rha, Ara, Xyl, and Gal were the major monosaccharides, accounting for more than 80% of the total monosaccharides, reaching even up to 90% for CAP. Compared to CAP, CAP-C exhibited significant changes in monosaccharide composition, with a significant increase in the content of Rha (6.37%) and Glc (11.05%) (*p* < 0.05), and a significant decrease in the content of Xyl (−9.85%) and Gal (−8.83%) (*p* < 0.05). Both CAP-A and CAP-S showed characteristic changes of elevated Glc content (6.02 and 5.68%), accompanied by decreased Xyl content (−6.09% and −6.40%); CAP-P demonstrated a distinct variation pattern, characterized by increased contents of Glc (6.57%) and Gal (9.52%), along with significant reductions in Rha (−8.16%) and Xyl (−8.11%) (*p* < 0.05). Notably, all derivatives retained the monosaccharide type of the natural polysaccharide, despite the altered monosaccharide ratio, suggesting that the modification process did not disrupt the basic backbone structure of the polysaccharide (71). Based on these findings, it can be inferred that all modification reactions may preferentially target specific types of sugar residues, or exhibit selective modification characteristics at different hydroxyl sites of the same sugar residue (74). This finding is consistent with the results reported by Abuduwaili et al. (31) and further confirms the differential reactivity exhibited by distinct monosaccharide residues during modification reactions.

#### 3.5.2 UV and FT-IR analysis

**Figure 5:**
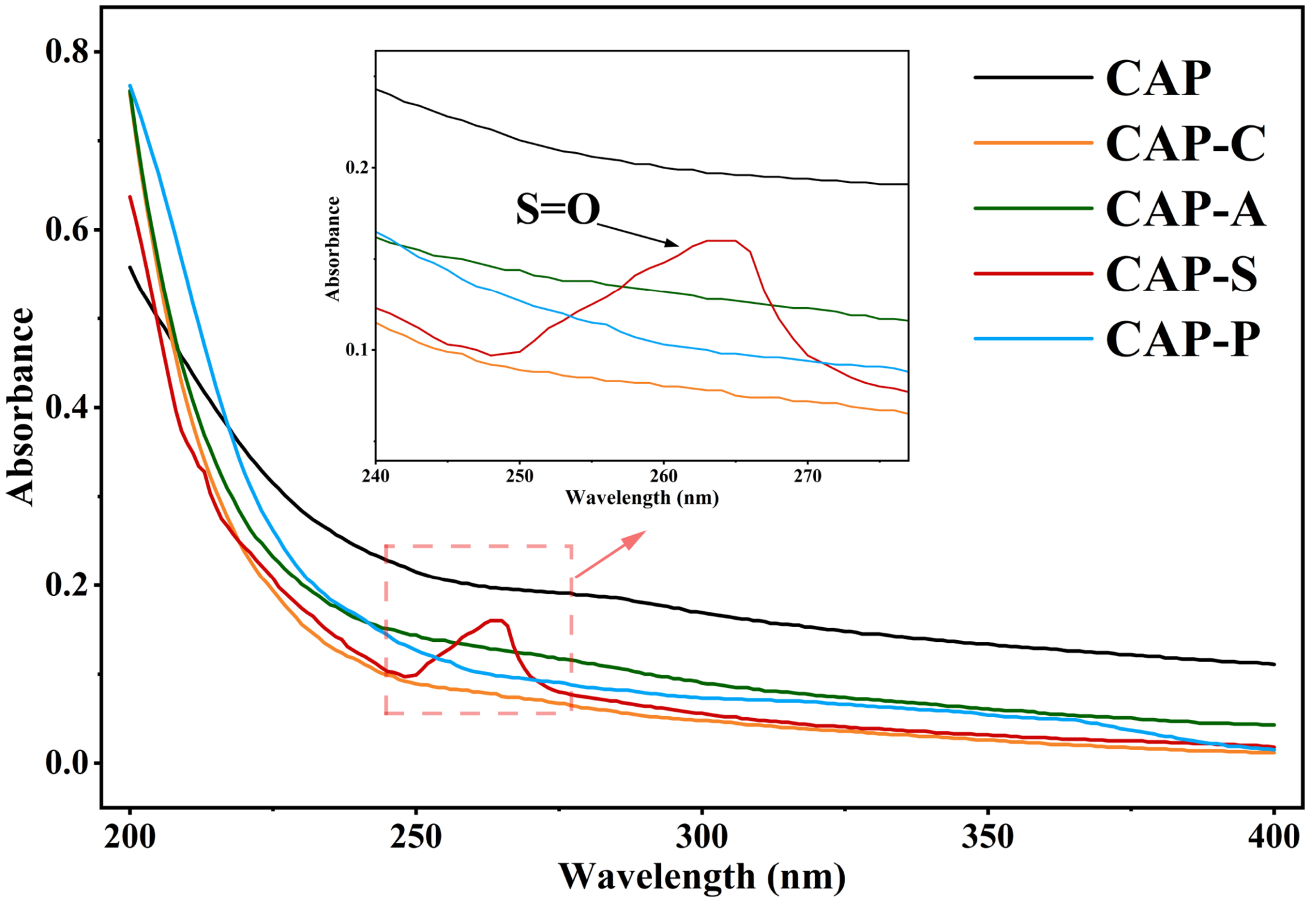
UV spectra of CAP and its derivatives.

The UV spectra of CAP and its derivatives are shown in Figure 5. There is an obvious absorption peak at 280 nm for CAP, while the rest of the derivatives exhibit a weaker absorption in this region, indicating that all the samples contain trace amounts of protein components (58). In addition, CAP-S displays a significant peak at 260 nm, which is attributed to the n → π* electron jump of the sulfate group (S=O) (42). This finding is consistent with the previous results of Cao et al. (75) and further confirms the success of the sulfation modification.

**Figure 6:**
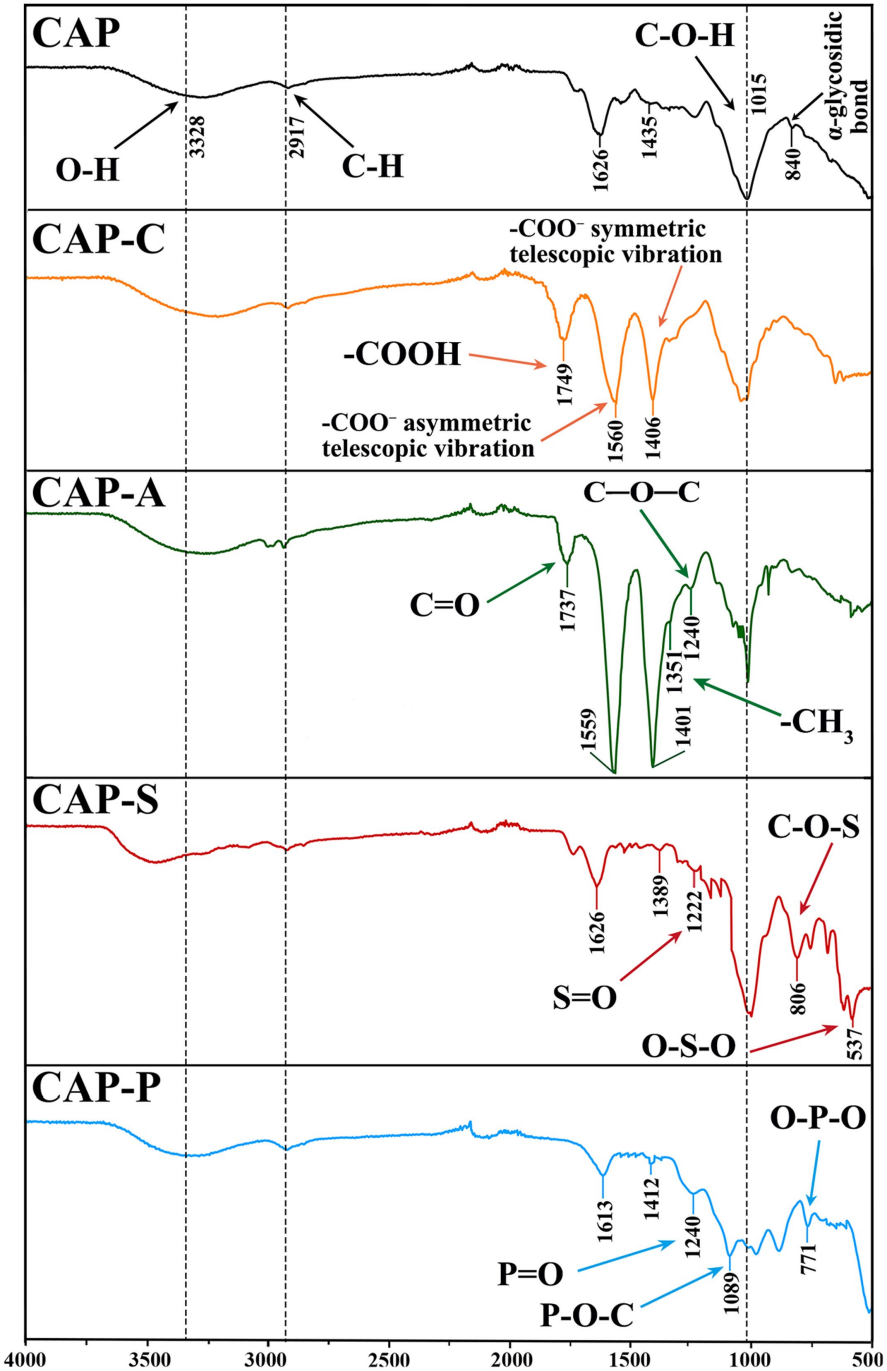
FT-IR spectra of CAP and its derivatives.

FT-IR is a pivotal technique for the structural characterization of polysaccharides. Figure 6 shows the FT-IR spectra of CAP and its derivatives. All polysaccharides exhibited characteristic absorption peaks within the wavenumber range of 4,000 ~ 500 cm^−1^. Specifically, the broad absorption peak at 3328 cm^−1^ corresponds to the O-H stretching vibration of sugar molecules, while the peak at 2,917 cm^−1^ is attributed to the C-H stretching vibrations of CH₂ and CH₃ groups (41). The absorption band observed at approximately 1,600 cm^−1^ could be assigned to the bending mode of water molecules (O-H deformation) or to the protein amide I vibrations (C=O stretching) (58). The peak near 1,400 cm^−1^ reflects the symmetric stretching vibration of deprotonated -COO^−^ groups, while the strong absorption bands in the range of 1,200 ~ 1,000 cm^−1^ represent the characteristic polysaccharide fingerprint region, primarily arising from C-O-C and C-O-H stretching vibrations (40). Furthermore, the characteristic peak at 840 cm^−1^ confirms the presence of α-glycosidic bonds in CAP (30), a finding consistent with both ^1^H and ^13^C NMR analyses.

Chemical modifications introduced characteristic absorption peaks into the natural polysaccharide structure (42). Compared with CAP, CAP-C exhibits a stretching vibration peak for the −COOH group at 1,749 cm^−1^, whilst new absorption peaks appear at 1560 cm^−1^ and 1,407 cm^−1^, which are attributed to the asymmetric stretching vibration and symmetric stretching vibration of the −COO^−^ group, respectively (76). CAP-A displayed a strong absorption peak at 1,737 cm^−1^, assigned to the C=O bond stretching vibration of the ester carbonyl group, along with symmetric bending vibrations of the -CH₃ group at 1,351 cm^−1^ and stretching vibrations of the C-O-C bond at 1,240 cm^−1^ (22). The presence of these characteristic peaks confirmed the successful incorporation of acetyl groups. The CAP-S showed an S=O asymmetric stretching vibration peak at 1,222 cm^−1^ (75). The absorption peak at 806 cm^−1^ originated from the asymmetric stretching vibration of the C-O-S bond, while the characteristic peak at 573 cm^−1^ corresponded to the symmetric stretching vibration of the O-S-O bond (23). These results indicate that the hydroxyl group was successfully converted into an O-sulfate group, confirming the successful sulfation modification. The new band at 1,240 cm^−1^ in CAP-P is attributed to the asymmetric stretching vibration of the P=O bond in the phosphorylated derivative (60). Additionally, the absorption peak at 1,089 cm^−1^ results from the stretching vibration of the P-O-C bond, while the peak at 771 cm^−1^ corresponds to the bending vibration of the O-P-O bond (24).

#### 3.5.3 Congo red analysis

**Figure 7:**
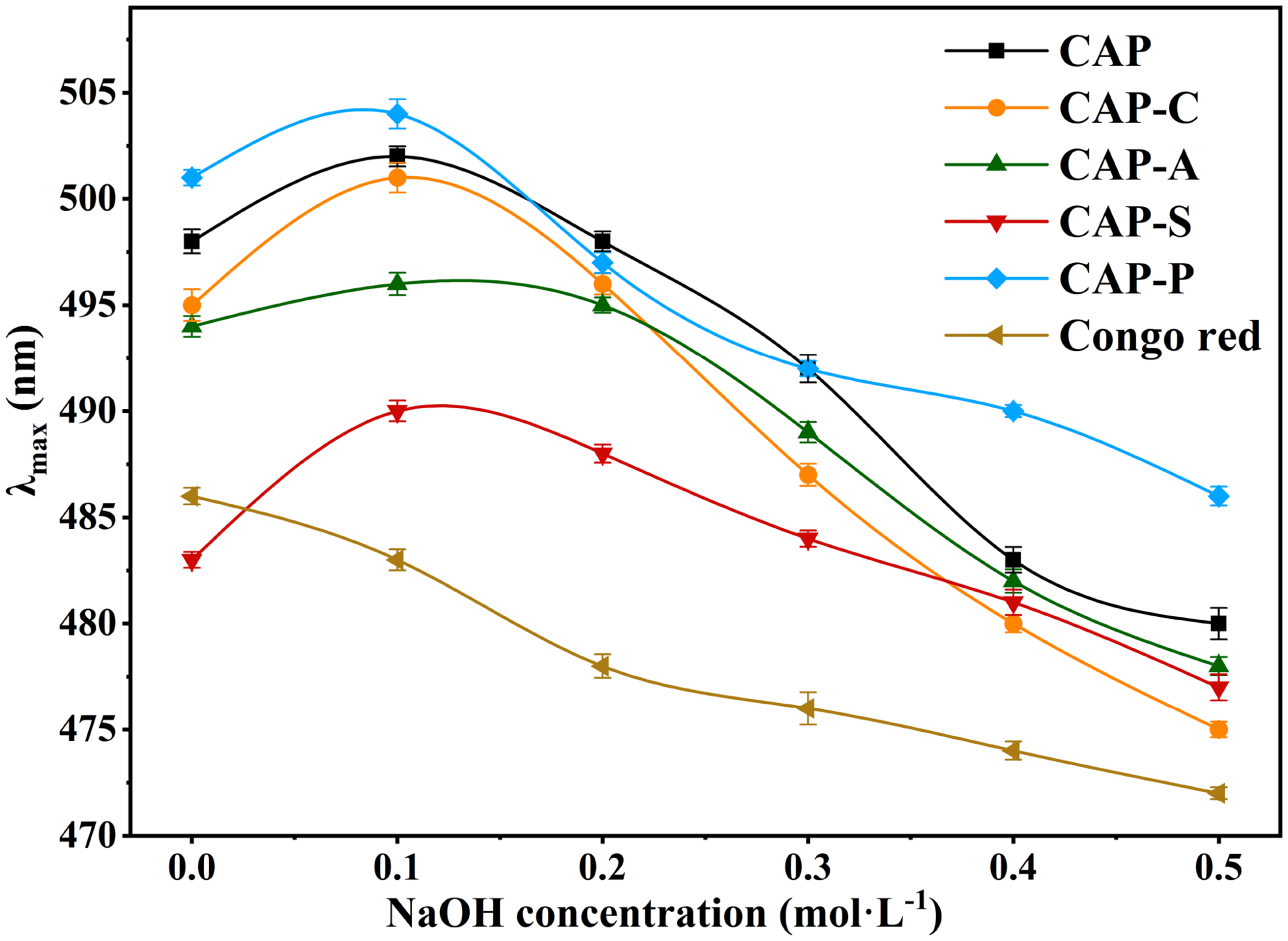
Variation in the λ_max_ of Congo red-polysaccharide complexes in NaOH solutions of different concentrations.

In low-concentration NaOH solutions, polysaccharides with an ordered helical conformation form complexes with Congo red, leading to a red shift in the λ_max_ (40). However, with increasing NaOH concentration, structural changes such as deconvolution and irregular curling of polysaccharides may result, causing a blue shift in λ_max_ (42). As shown in Figure 7, the λ_max_ of both CAP and its derivatives showed a concentration-dependent trend in the range of 0.1 ~ 0.5 mol/L NaOH, with a red shift followed by a blue shift, when compared to the control group (Congo red group). This observation confirms the existence of a stable triple-helical structure in these polysaccharides. Notably, all four polysaccharide derivatives exhibited trends similar to those of natural polysaccharides, indicating that the structural modifications did not alter the fundamental structure of the polysaccharide backbone (71). Ren et al. (77) demonstrated that both carboxymethylation and acetylation modifications preserved the triple-helical conformation of *Cordyceps militaris* polysaccharides, consistent with our current findings. However, their study did not investigate the effects of sulfation or phosphorylation on this tertiary structure.

#### 3.5.4 SEM analysis

**Figure 8:**
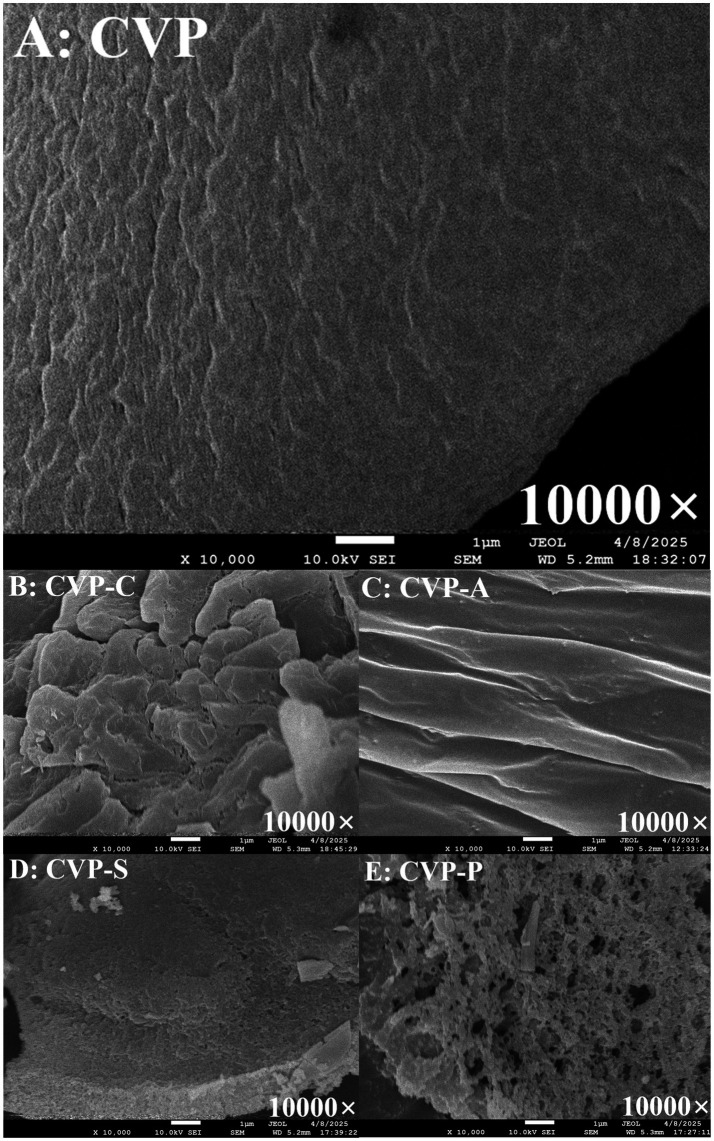
SEM images of the CAP **(A)**, CAP-C **(B)**, CAP-A **(C)**, CAP-S **(D)**, and CAP-P **(E)** at 10,000× (scale bar: 1 μm).

In order to systematically characterize the effect of chemical modifications on the morphology of polysaccharides, we observed the morphology of CAP and its derivatives at 10,000× magnification. As shown in Figure 8, CAP displays an irregular lamellar morphology, characterized by a textured yet compact surface and distinct edges (Figure 8A). The polysaccharide morphology underwent significant changes following chemical modifications. Notably, CAP-C exhibited a rough and loose surface with a highly porous architecture, in which interconnected macropores formed a complex network (Figure 8B). This structure facilitated water molecule penetration, thereby enhancing polysaccharide solubility (35). These findings align with observations by Zhang et al. (78) on *Pholiota nameko* polysaccharide, where porous structures similarly promoted water infiltration and solubility improvement. Compared to CAP, the surface of CAP-A was smoother, exhibiting thin, film-like, and lamellar structures with fine particle aggregates (Figure 8C). This morphology might result from an increased degree of cross-linking between polysaccharide molecules (79). In contrast, CAP-S displayed a blocky structure with a rough surface (Figure 8D), which is highly similar to the microstructure of sulfated polysaccharides from *Sargassum pallidum* reported by Xiao et al. (80). This phenomenon can be attributed to the sulfate groups disrupting hydrogen bonds in the polysaccharides, leading to disordered molecular arrangements and the formation of irregular, block-like structures (73). The surface of CAP-P exhibited a rough, spongy morphology, with an overall looser structure and a significantly increased specific surface area (Figure 8E). This resembled the morphology of phosphorylated *Asarum sieboldii* polysaccharides (81), likely resulting from the combined effects of enhanced cross-linking between polysaccharide molecules and high-temperature-induced conformational changes.

#### 3.5.5 XRD analysis

**Figure 9:**
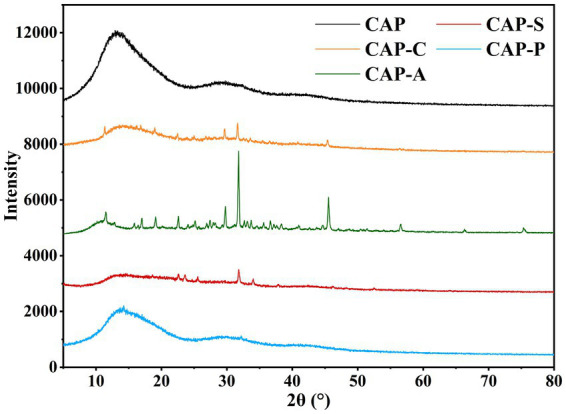
XRD analysis of the CAP and its derivatives.

XRD is a key technique for resolving the crystallinity of polysaccharides. As shown in Figure 9, CAP and its derivatives exhibit distinct crystallization properties. CAP and CAP-P displayed broadened diffraction peaks near 13°, 29°, and 41°, along with diffuse scattering regions, indicating predominantly amorphous structures with minor crystalline domains. This phenomenon may be due to incomplete hydrogen bonding between polysaccharide molecules, resulting in the presence of many free hydroxyl groups (42). In contrast, CAP-C and CAP-S exhibited weak diffraction peaks at 22°, 25°, and 31°, while retaining amorphous characteristics near 14°, suggesting a coexistence of microcrystalline and amorphous phases in the carboxymethylated and sulfated derivatives (73). Notably, CAP-A showed sharp, intense peaks at 11°, 29°, 31°, and 45°, confirming a highly ordered crystalline state (34).

#### 3.5.6 Thermal property analysis

**Figure 10:**
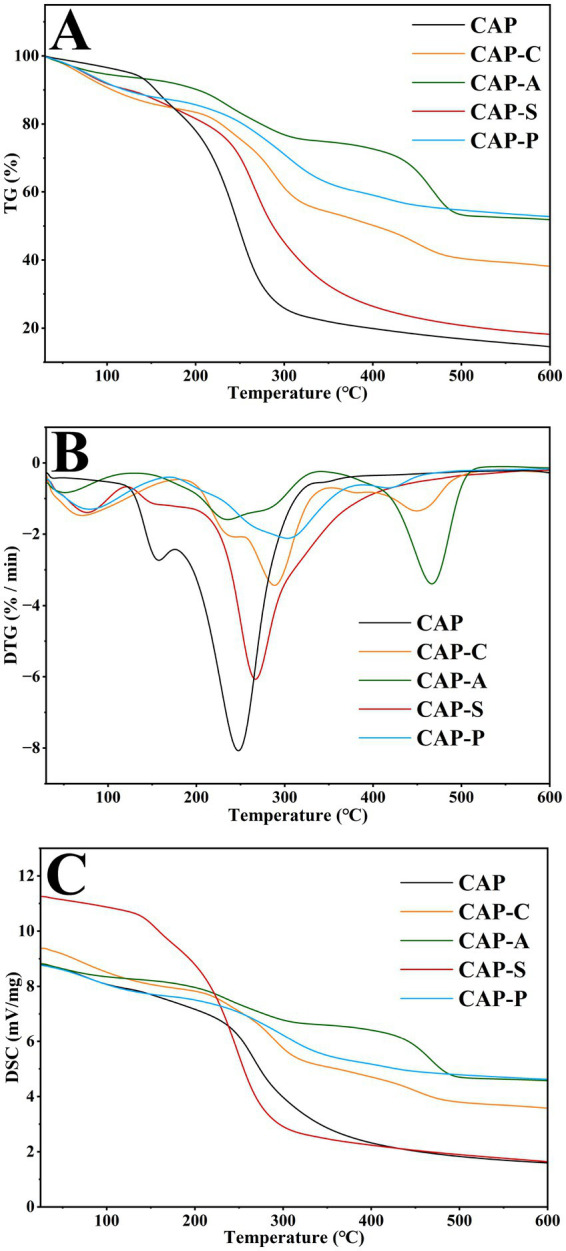
Thermal properties of CAP and its derivatives. TG curve **(A)**, DTG curve **(B)**, and DSC curve **(C)**.

The thermal properties of polysaccharides directly affect their applications in the food and pharmaceutical fields. The thermal properties of CAP and its derivatives were evaluated by TG, DTG, and DSC. The TG curves (Figure 10A) revealed that both CAP and its derivatives underwent three distinct mass loss stages in the range of 30 °C ~ 600 °C. The first stage (32 °C ~ 142 °C) corresponded to the evaporation of bound and free water from the polysaccharides (79). The second stage (201 °C ~ 316 °C) exhibited a mass loss exceeding 50% of the initial weight, primarily due to the degradation of the polysaccharide backbone (40). The third stage (327 °C ~ 600 °C) demonstrated a slower degradation rate prior to reaching a stable state. The final residual masses in descending order were: CAP-P (52.87%) > CAP-A (51.77%) > CAP-C (39.84%) > CAP-S (18.37%) > CAP (14.04%). DTG analysis (Figure 10B) showed that the maximum decomposition rates of CAP, CAP-C, CAP-A, CAP-S, and CAP-P were 8.07%/min (247.29 °C), 3.14%/min (287.45 °C), 3.41%/min (465.16 °C), 6.06%/min (267.09 °C), and 2.11%/min (305.81 °C), respectively. Notably, all derivatives exhibited lower maximum decomposition rates and higher degradation temperatures compared to CAP. Carboxymethylation, acetylation, sulfation, and phosphorylation modifications were confirmed to enhance the thermal stability of polysaccharides. Meanwhile, the DSC results (Figure 10C) showed that CAP and its derivatives exhibited a peak between 150.47 °C and 271.69 °C, which was related to the glass transition temperature of the polysaccharide (42). As the temperature increased, another peak appeared at 346 °C ~ 480 °C, which may have been caused by polysaccharide degradation (31). By comparing the TG, DTG, and DSC curves, it was evident that CAP remained relatively stable up to 170 °C, while its derivatives remained relatively stable up to 210 °C.

### 3.6 Anti-inflammatory activity analysis

#### 3.6.1 Cytotoxicity of CAP and its derivatives on RAW264.7 cells

**Figure 11:**
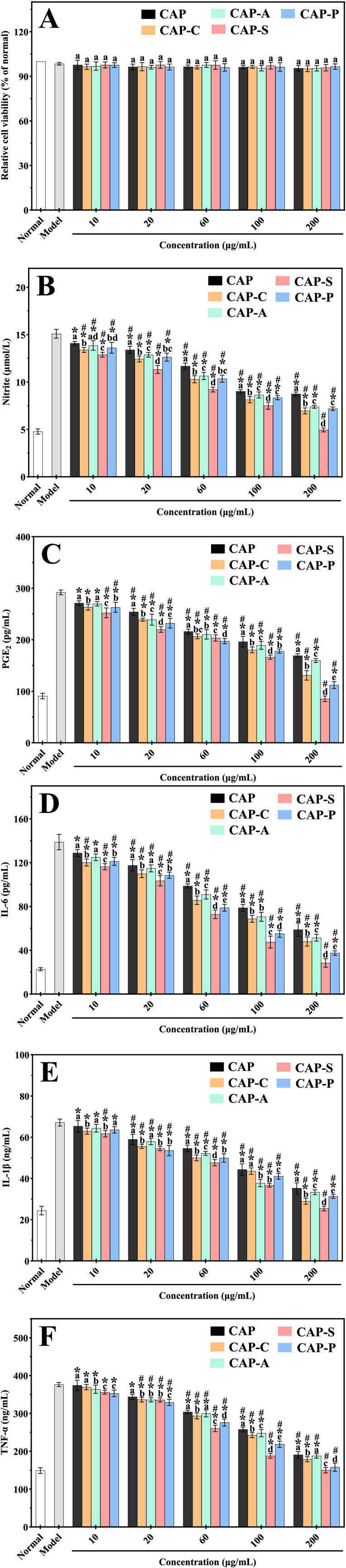
Cytotoxic effects of CAP and its derivatives on RAW264.7 macrophages **(A)**. Inhibitory effects of CAP and its derivatives on the production of NO **(B)**, PGE_2_ **(C)**, IL-6 **(D)**, IL-1β **(E)**, and TNF-α **(F)** in LPS-induced RAW264.7 macrophages. Different lowercase letters **(A–E)** indicate significant differences between samples at the same concentration (*p* < 0.05). “*” indicates a significant difference compared with the normal group (*p* < 0.05). “#” indicates a significant difference compared with the model group (*p* < 0.05).

As shown in Figure 11A, within the concentration range of 10 ~ 200 μg/mL, neither CAP nor its derivatives exhibited significant changes in cell viability compared to the normal group (*p* > 0.05). Notably, the cell viability of the model group (98.42 ± 0.64%) also showed no statistically significant difference compared to the normal group (100%) (*p* > 0.05). Additionally, no significant differences were observed between CAP and its derivatives at the same concentrations (*p* > 0.05). The results indicate that neither CAP and its derivatives nor LPS had cytotoxic effects on RAW264.7 macrophages.

#### 3.6.2 Impact of CAP and its derivatives on LPS-induced NO/PGE2 production

LPS-induced NO and PGE_2_ are two key bioactive molecules in inflammatory responses. As an important signaling molecule in various cells, the excessive production of NO is closely associated with the pathogenesis of multiple inflammatory diseases (44). Therefore, detecting NO levels within tissues or cellular microenvironments provides a direct assessment of cellular functional status and the anti-inflammatory effects of relevant drugs. Similarly, PGE_2_, a lipid mediator produced through the cyclooxygenase (COX)-mediated metabolism of arachidonic acid, participates in cellular signaling processes such as inflammatory responses and tumor metastasis (4). As shown in Figures 11B,C, compared with the normal group, the levels of NO and PGE_2_ in LPS-treated RAW264.7 macrophages (model group) were significantly increased (*p* < 0.05), indicating the successful establishment of the inflammatory cell model. After treatment with CAP and its derivatives, the levels of NO and PGE₂ in the cells decreased in a concentration-dependent manner, with significant differences in the inhibitory effects among the derivatives at the same concentration (*p* < 0.05). Among them, within the concentration range of 10 ~ 200 μg/mL, CAP-S exhibited the highest inhibition rate on NO (67.22 ± 0.38%), followed by CAP-C (53.84 ± 0.25%), CAP-P (52.45 ± 0.21%), CAP-A (51.06 ± 0.17%), and CAP (42.05 ± 0.14%). A similar trend was observed for PGE_2_ inhibition, with CAP-S (70.71 ± 0.48%) showing the most potent effect, followed by CAP-C (61.63 ± 0.45%), CAP-P (55.12 ± 0.53%), CAP-A (45.25 ± 0.22%), and CAP (42.07 ± 0.28%). The results indicate that the chemically modified derivatives inhibited both NO and PGE_2_ more effectively than the natural polysaccharides, with the sulfated modification being the most effective.

#### 3.6.3 Impact of CAP and its derivatives on LPS-induced proinflammatory cytokine production

The excessive production of pro-inflammatory cytokines (IL-6, IL-1β, and TNF-α) by immune cells can induce severe inflammation-related diseases in RAW264.7 macrophages. Among these, IL-6 is an endogenous pyrogenic mediator induced by LPS, which is responsible for regulating the production of acute phase proteins (82). IL-1β is released during the early stages of inflammation and is closely associated with various inflammatory diseases (83). TNF-α can enhance immune cell activity and promote the synthesis and release of other inflammatory factors (84). To investigate the anti-inflammatory effects of CAP and its derivatives on LPS-induced RAW264.7 macrophages, we measured the secretion levels of these three key pro-inflammatory cytokines, which are closely associated with the production of NO and PGE_2_. As shown in Figures 11D–F, in the LPS-induced RAW264.7 macrophage inflammation model, the levels of IL-6, IL-1β, and TNF-α were significantly upregulated by 2.52 ~ 6.11 fold compared to the normal group (*p* < 0.05). This indicates that LPS triggers a severe inflammatory response by disrupting the balance of these cytokines within the NF-κB/MAPK signaling pathway (41). After treatment with CAP and its derivatives, the secretion of these cytokines was significantly inhibited, with significant differences in the inhibitory effects among the derivatives at the same concentration (*p* < 0.05). Within the concentration range of 10 ~ 200 μg/mL, compared to the model group, the order of IL-6 reduction in RAW264.7 macrophages by treatment with CAP and its derivatives was (Figure 11D): CAP-S (79.52 ± 0.58%) > CAP-P (72.99 ± 0.56%) > CAP-C (65.42 ± 0.43%) > CAP-A (62.92 ± 0.46%) > CAP (57.59 ± 0.34%); in terms of IL-1β inhibition (Figure 11E): CAP-S (62.14 ± 0.42%) > CAP-C (56.83 ± 0.35%) > CAP-P (53.35 ± 0.41%) > CAP-A (50.31 ± 0.29%) > CAP (47.47 ± 0.33%); in terms of TNF-α inhibition (Figure 11F): CAP-S (60.11 ± 0.37%) > CAP-P (58.01 ± 0.44%) > CAP-C (52.44 ± 0.24%) > CAP-A (50.02 ± 0.31%) > CAP (49.28 ± 0.26%). Overall, the levels of IL-6, IL-1β, and TNF-α in RAW264.7 macrophages treated with the modified derivatives were significantly lower than those in cells treated with the natural polysaccharide (*p* < 0.05), indicating that the modified polysaccharides possess stronger anti-inflammatory activity. Notably, CAP-S exhibited the strongest inhibitory effect on all three inflammatory factors, which may be attributed to the introduced sulfate groups enhancing the negative charge characteristics of the polysaccharide, thereby more effectively blocking pro-inflammatory signaling pathways (26).

## 4 Conclusion

In this study, the extraction process of polysaccharides from *C. arenarius* L. was optimized, and a high-purity polysaccharide fraction, CAP, was successfully isolated and purified, with a molecular weight of 286.49 kDa. Through comprehensive studies, including partial acid hydrolysis, Smith degradation, methylation analysis, and NMR spectroscopy, it was determined that its backbone consists of sugar residues derived from xylose (Xyl), mannose (Man), glucose (Glc), and galactose (Gal). The structure features multiple linkage types, including →3)-Xyl*p*-(1→, →4)-Xyl*p*-(1→, Man*p*-(1→, →3,6)-Man*p*-(1→, Glc*p*-(1→, →6)-Glc*p*-(1→, →3)-Gal*p*-(1→, Gal*p*-(1→, and →2,4)-Gal*p*-(1→. The terminal residues are primarily composed of T-Rha*p*, T-Ara*f*, and T-Xyl*p*. Various analytical techniques, such as UV, FT-IR, Congo red assay, SEM, XRD, TG, DTG, and DSC, revealed that the four derivatized polysaccharides possessed significantly different physicochemical properties and structural characteristics. Anti-inflammatory experiments showed that treatment of RAW264.7 macrophages with CAP and its derivatives effectively suppressed inflammatory responses, reduced NO and PGE_2_ production, and decreased the secretion of pro-inflammatory cytokines (IL-6, IL-1β, and TNF-α), with CAP-S exhibiting the strongest anti-inflammatory activity. The above results suggest that CAP and its derivatives have the potential to be developed as novel foods with immunomodulatory functions. However, subsequent studies still need to combine signal pathway regulation, TLR4 antibody blockade, and inflammation animal model experiments to elucidate the differences in the mechanisms of action of CAP and its derivatives, thereby further validating the conclusions of this study.

## Data Availability

The original contributions presented in the study are included in the article/Supplementary material, further inquiries can be directed to the corresponding author.
