# Supplementary material for: Extraction, structural characterization, chemical modification and anti-inflammatory activity of polysaccharides from Ceratocarpus arenarius L
Source: Front Nutr. 2026 Feb 26;13:1785022. doi: 10.3389/fnut.2026.1785022 (PMC12979405; doi:10.3389/fnut.2026.1785022)
Supplement: Supplementary file 1 [file Supplementary_file_1.docx]

Supplementary Material

# Supplementary Data

## Supplementary Figures

| 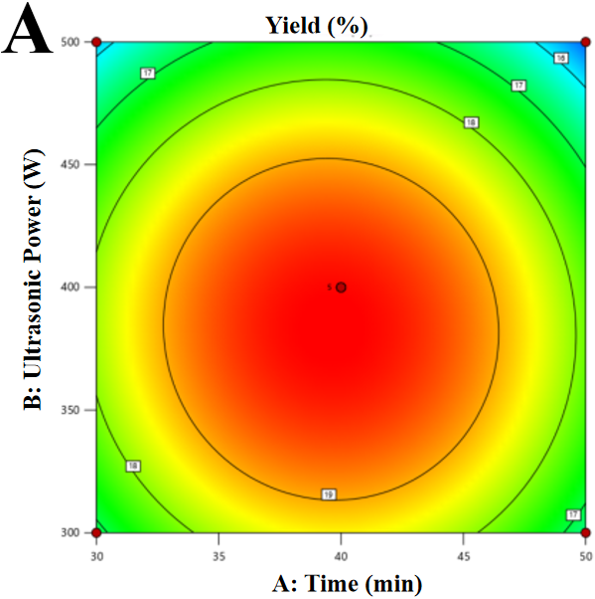 | 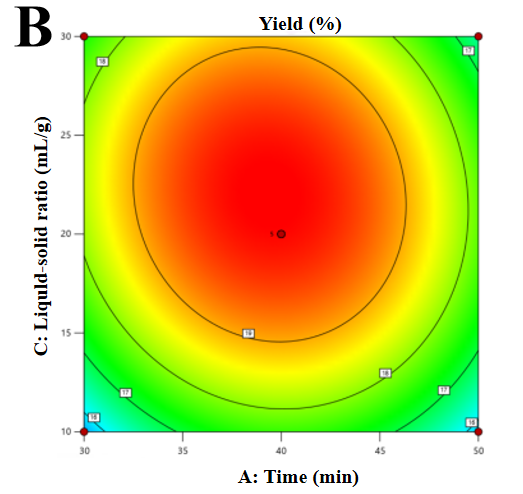 | 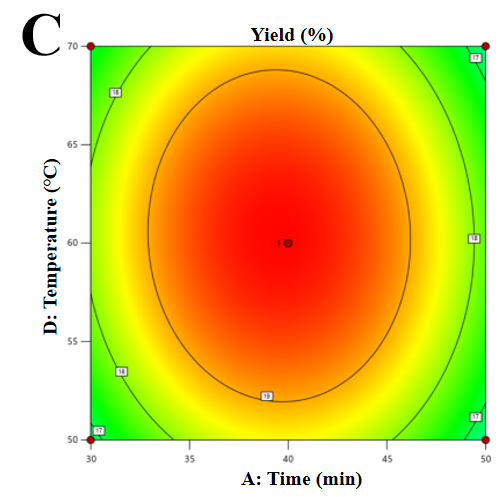 |
| --- | --- | --- |
| 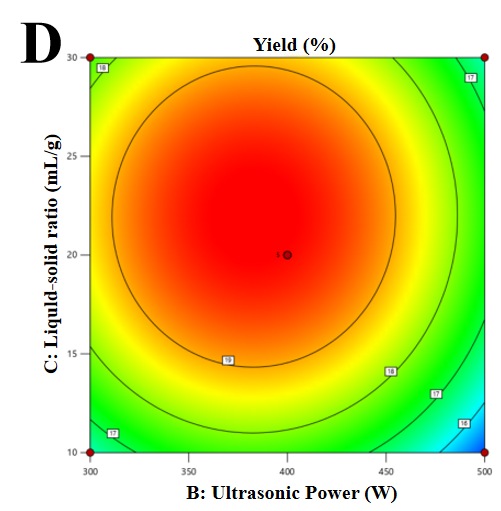 | 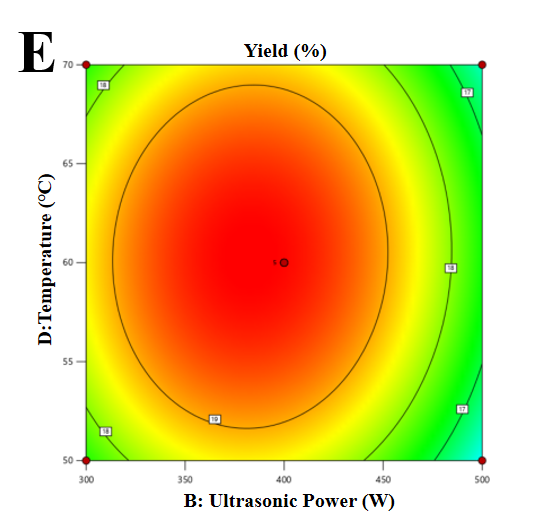 | 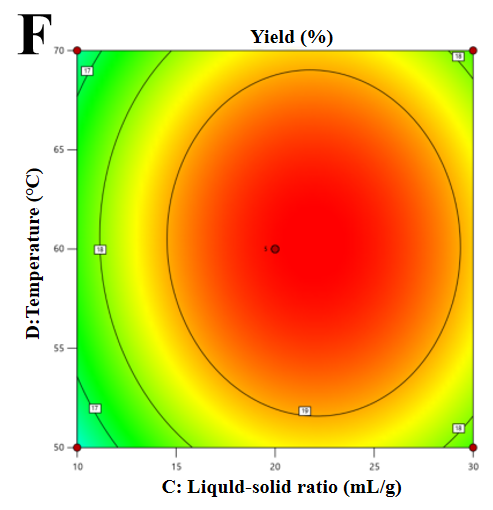 |

**Supplementary Figure 1.** Contour plots of factor interactions.

| 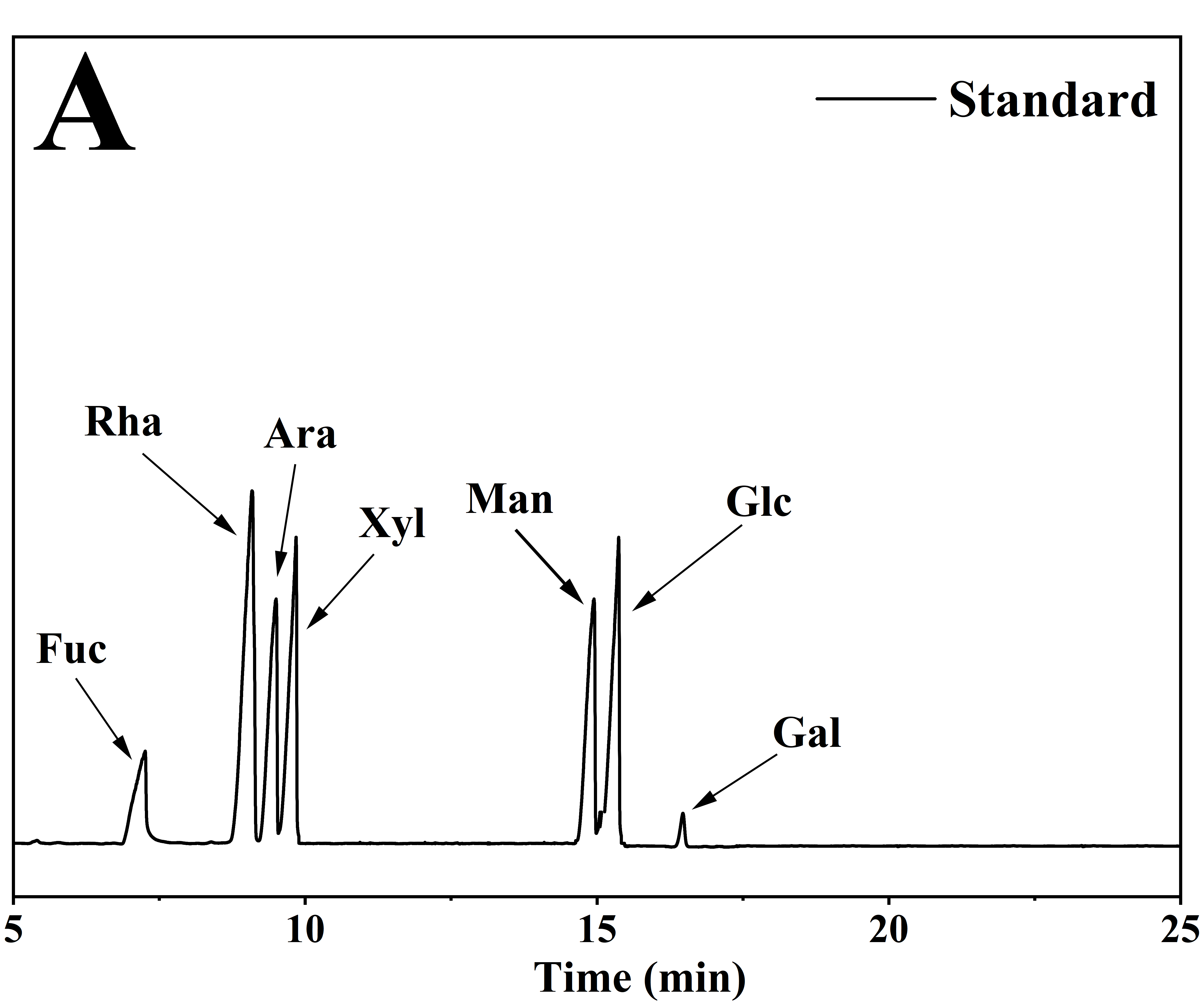 |
| --- |
| 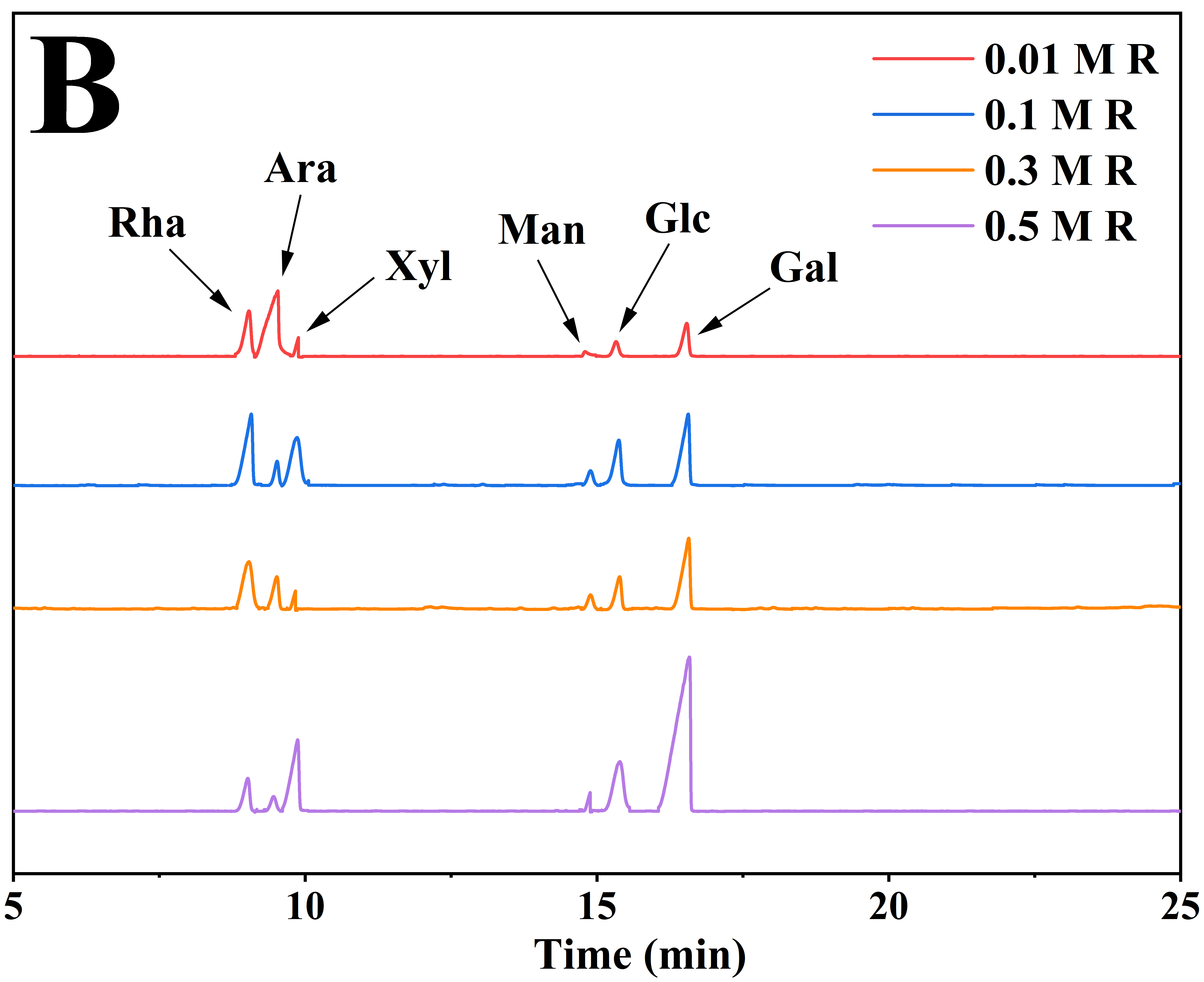 |
| 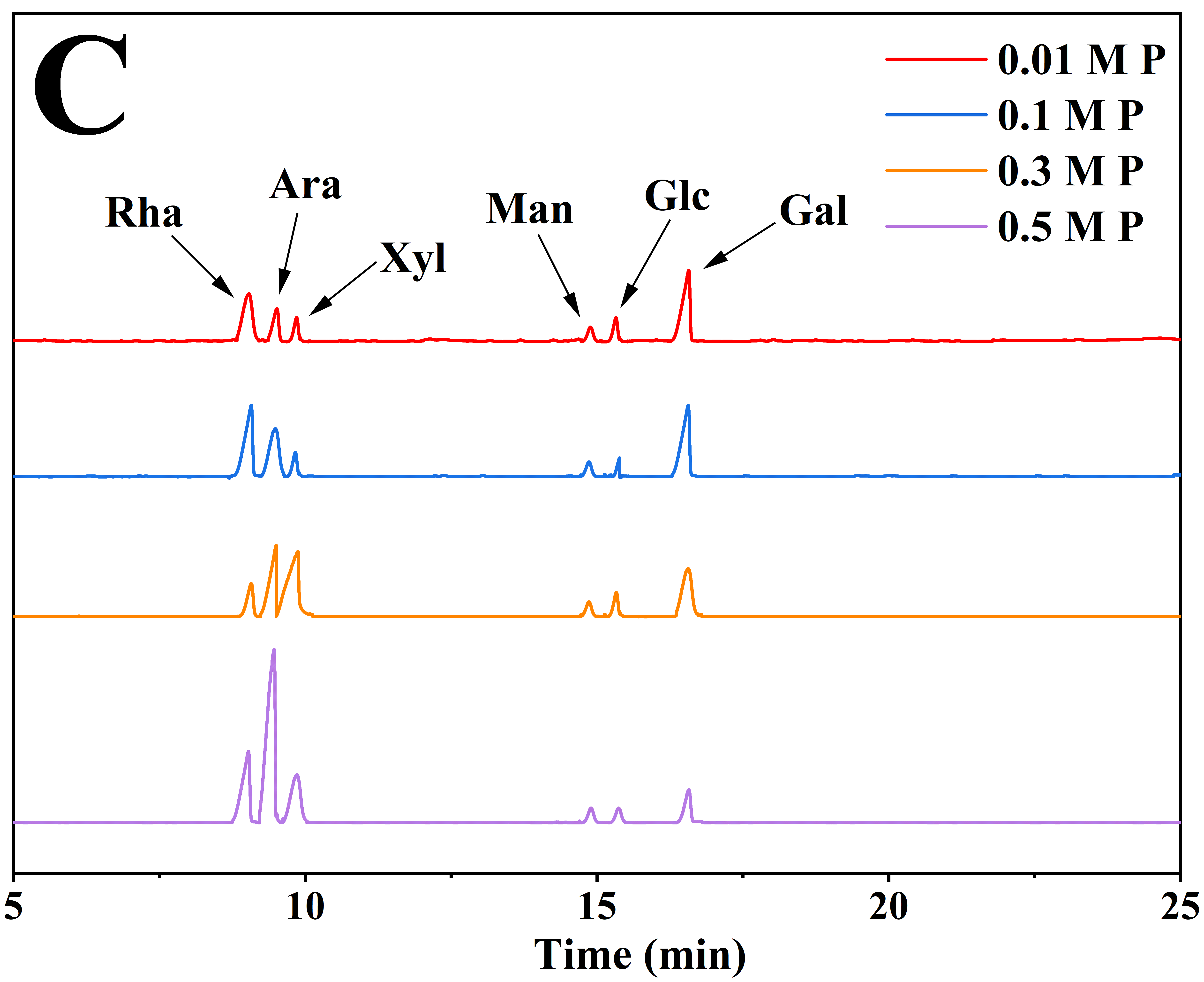 |

**Supplementary Figure 2.** GC spectra of the partial acid hydrolysis of CAP. Monosaccharides mixed standard (A), retentate part (B), and permeate part (C).


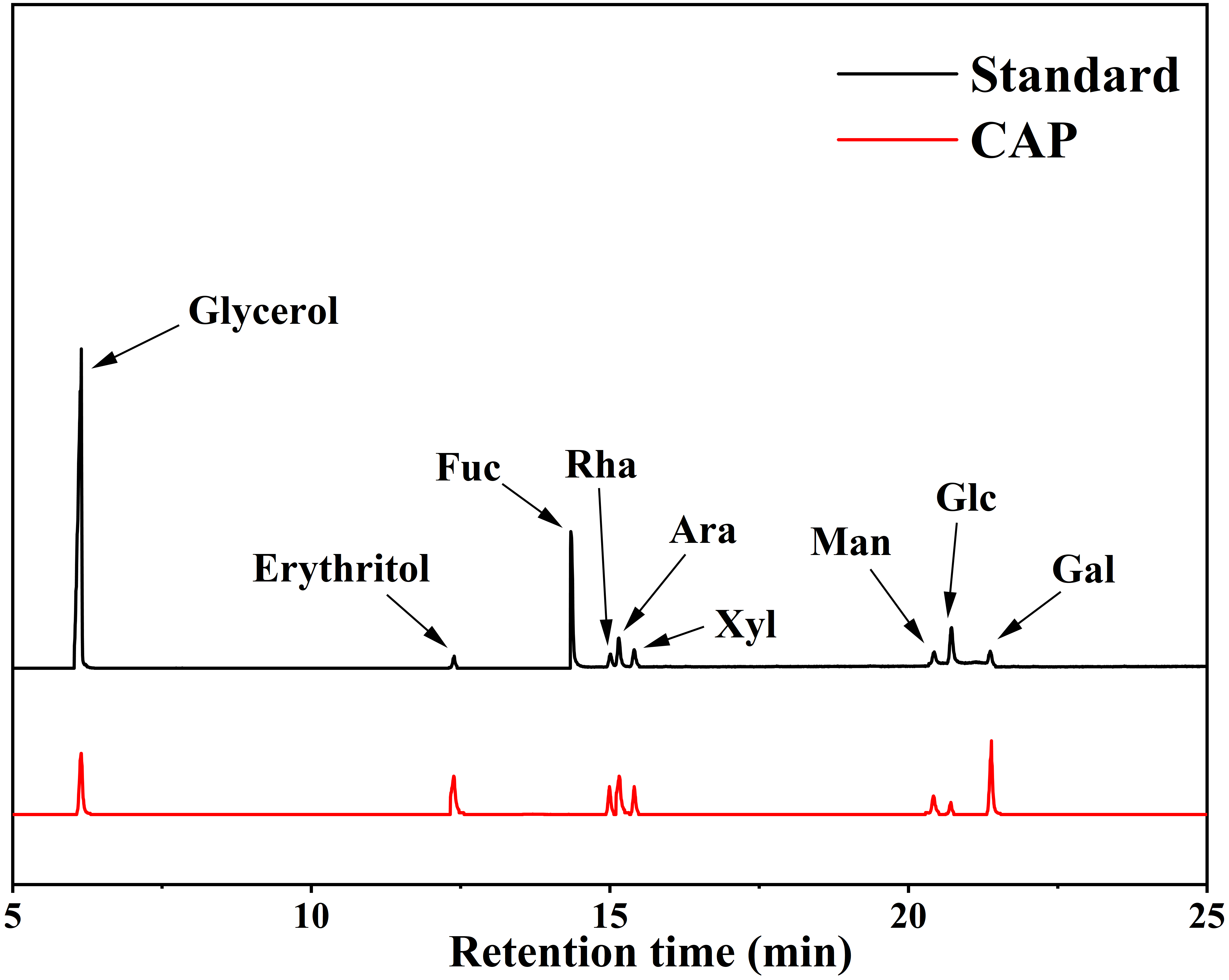


**Supplementary Figure 3.** GC spectra of the Smith degradation products of CAP.


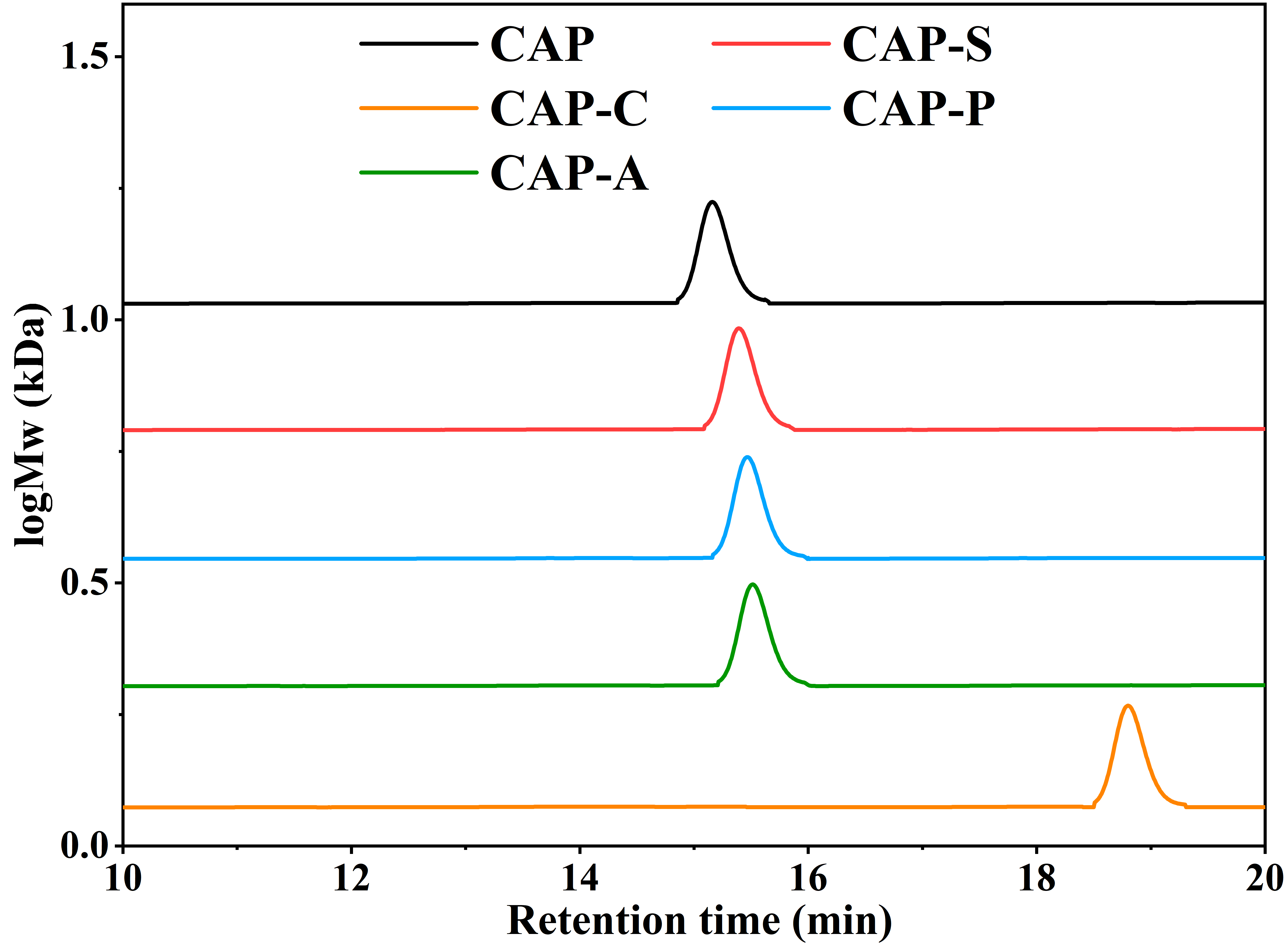


**Supplementary Figure 4.** Mw curves of CAP and its derivatives.

| 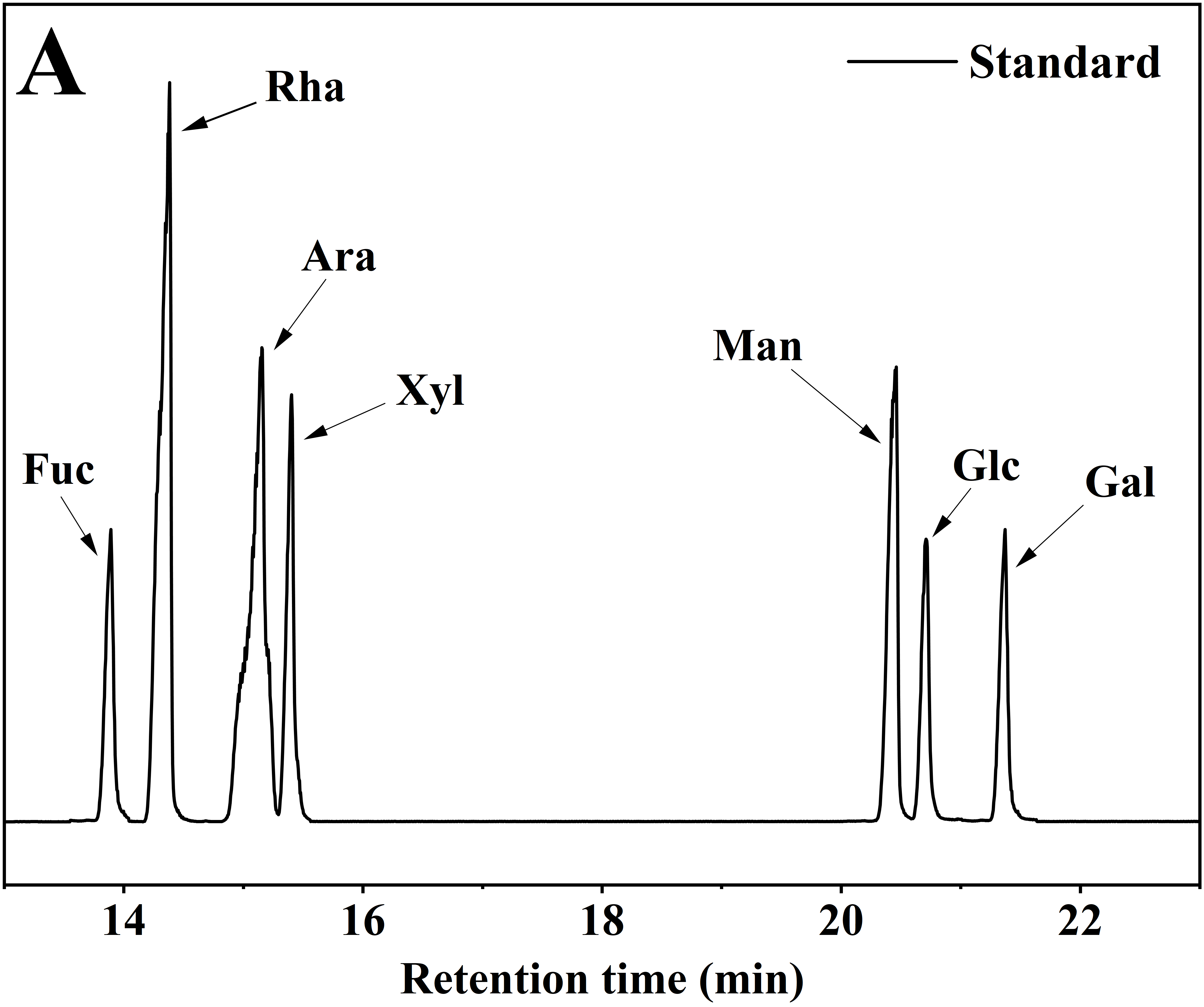 |
| --- |
| 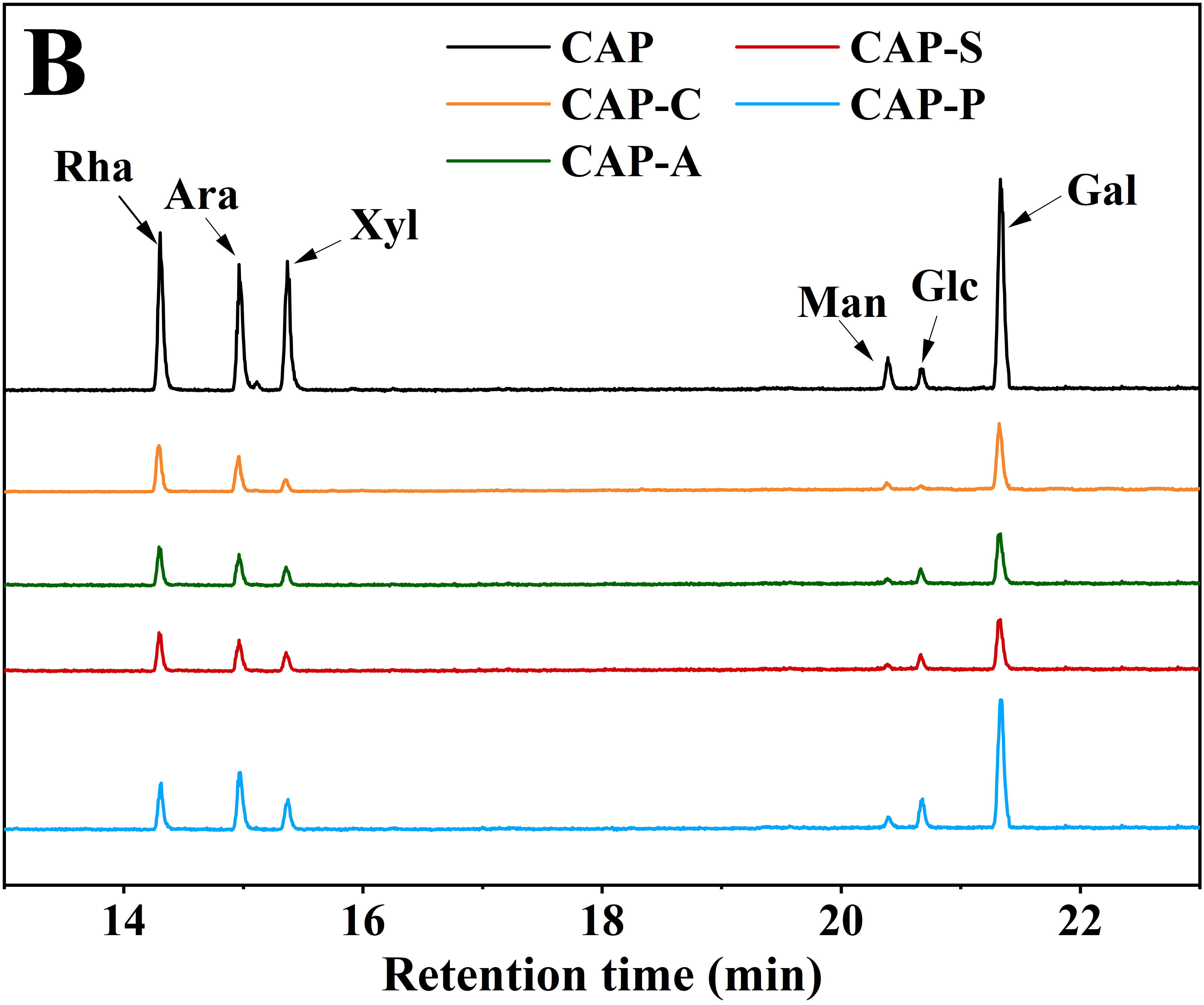 |

**Supplementary Figure 5.** Monosaccharide composition of CAP and its derivatives from GC.
